# Chemospecific cobalt-catalyzed hydroboration of CO_2_†

**DOI:** 10.1039/d5ra01656h

**Published:** 2025-06-03

**Authors:** Andrey Fedulin, Lea Luxenberger, Axel Jacobi von Wangelin

**Affiliations:** a Dept. of Chemistry, University of Hamburg Martin Luther King Platz 6 20146 Hamburg Germany axel.jacobi@uni-hamburg.de; b Dept. of Chemistry, University of Regensburg 93040 Regensburg Germany

## Abstract

A bifunctional cobalt pyridonate complex effectively catalyzes the hydroboration of CO_2_ to the boryl formate at very mild conditions (0.1–1 mol% cat., 1 bar CO_2_, r.t., 5 min, 100% yield, TON 1000, TOF 12 000 h^−1^). At higher temperature, clean conversion to the methoxyborane was achieved (98% yield). Mechanistic studies indicate formation of a ligand-derived cobalt hydride species.

## Abstract 2

A bifunctional cobalt pyridonate catalyzes the hydroboration of CO_2_ to boryl formate at mild conditions (<1 mol% cat., 1 bar CO_2_, r.t., 5 min, 100% yield, TOF 12 000 h^−1^). At higher temperature, clean conversion to the methoxyborane was achieved.

## Graphical abstract

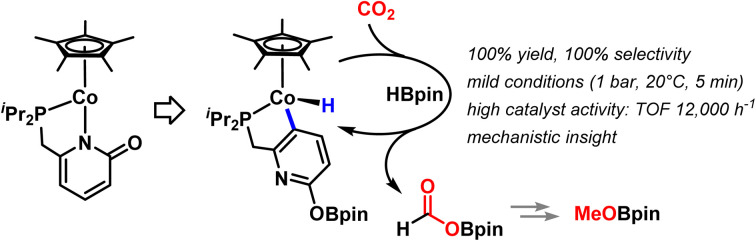

## Introduction

**Scheme 1:**
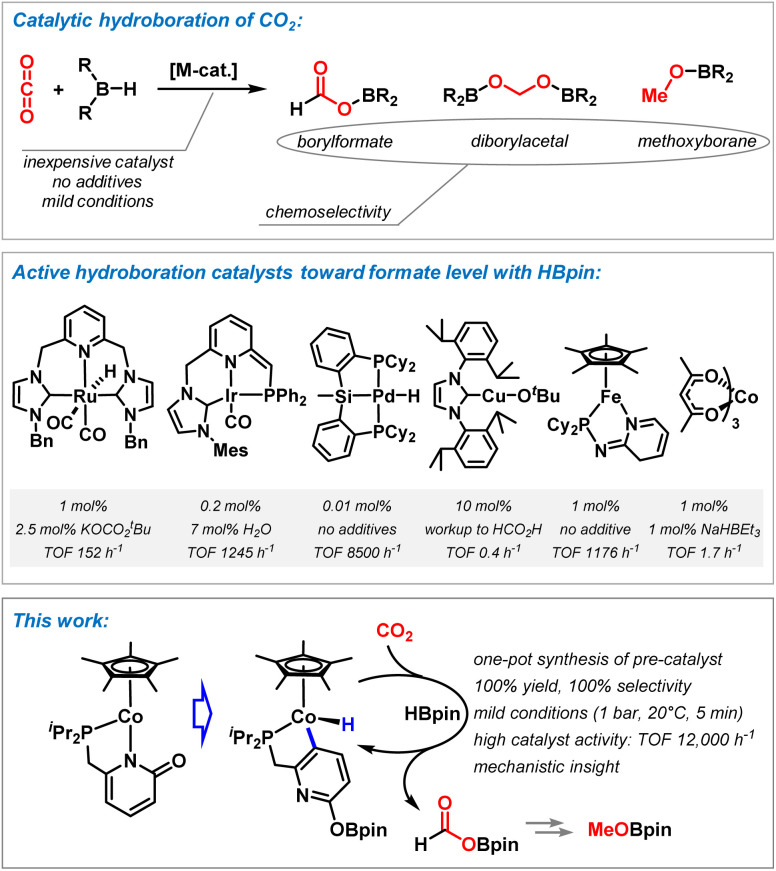
Top: metal catalyzed hydroboration of carbon dioxide. Middle: selected examples of active metal catalysts and reaction conditions. Bottom: Cobalt pyridonate catalyst for chemospecific CO_2_ hydroboration (= this work).

**Scheme 2:**
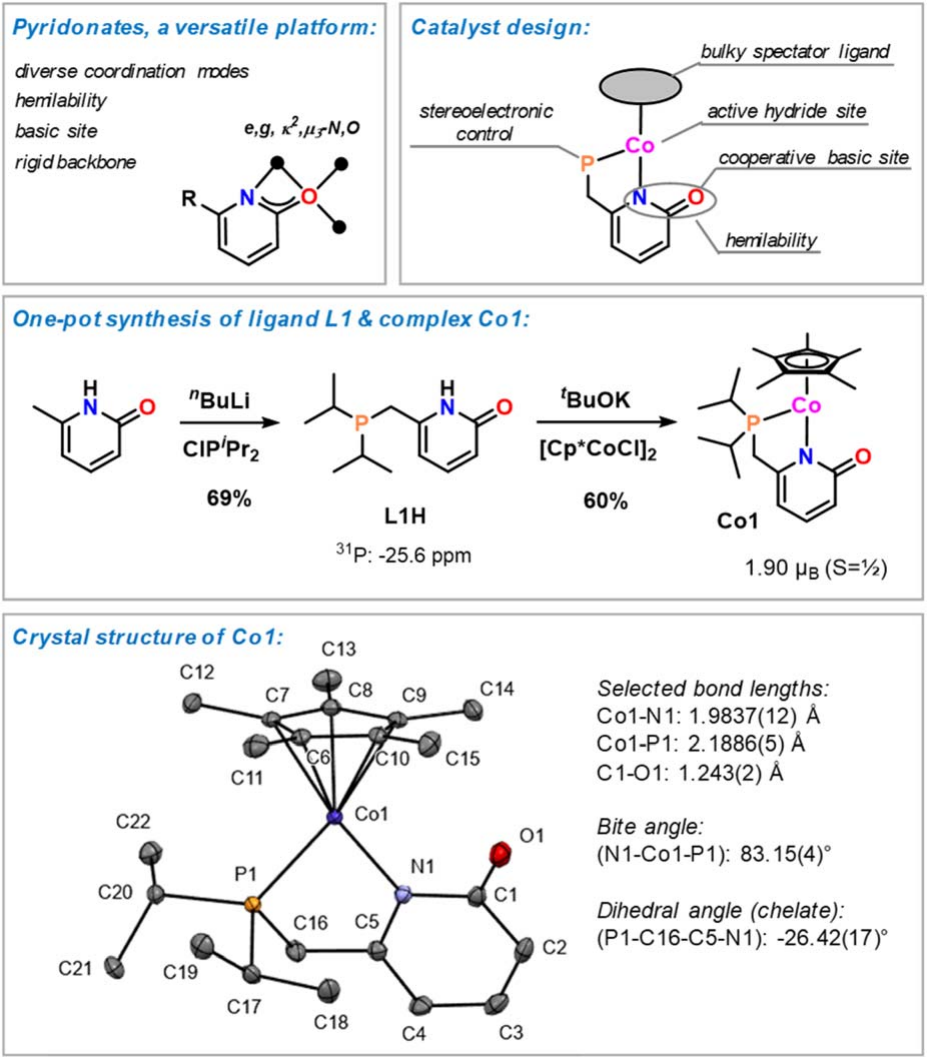
Design concept, synthesis, and structure of cobalt pyridonate Co1.^23^

Reductive transformations of carbon dioxide are key to all future scenarios in sustainable energy and chemical production technologies.^1,2^ The use of CO_2_ as chemical building block is strongly limited by its thermodynamic and kinetic stability, so that efficient catalytic mechanisms at mild conditions constitute a prime area of research. Various synthetic strategies of CO_2_ reduction have been developed to formate, formaldehyde, and methanol derivatives and methane. Technical processes mostly utilize hydrogenation reactions under high pressures of H_2_ and elevated temperatures, whereas lab-scale reactions often operate with more convenient liquid hydrogen surrogates such as boranes and silanes. Metal-catalyzed hydroborations of CO_2_ have been demonstrated to enable facile reductions to borylformates, diborylacetals, and methoxyboranes (Scheme 1, top).^3^ Major challenges reside in the highly selective formation of a single reduced C1 building block, the use of inexpensive yet highly reactive catalysts and reducing reagents, and the operation under mild conditions with no excess reagents and without waste formation. For example, the two-electron reduction of CO_2_ with boranes in the presence of metal catalysts provides *O*-boryl formates that constitute valuable formyl and formate building blocks. So far, pincer-ligand supported noble metal catalysts (Pd,^4,5^ Ir,^6^ Ru^7^) were among the most active (Scheme 2, middle).^8^ An NHC-copper alkoxide catalyst gave 85% formic acid after hydrolysis;^9^ ligand-coordinated zinc hydride catalysts afforded moderate activity.^10,11^ Recently, an iron catalyst with an anionic PN ligand showed very good activity.^12^ Further examples of Mn,^13,14^ Fe,^15^ and Ni^16,17^ catalysts are known for CO_2_ hydroborations to diborylacetal and borylmethanol. A single example of a cobalt-catalyzed reduction of CO_2_ selectively to boryl formate was reported, with low to moderate yields and little mechanistic insight.^18^

Pyridonate ligands entertain a rich coordination chemistry with most transition metals due to their flexible binding modes, hemilability, and potential metal–ligand cooperativity.^19^ We reasoned that such multi-functional behavior of metal pyridonates may be effectively exploited for CO_2_ hydro-borations by sequential BH bond splitting, CO_2_ coordination, and hydride transfer onto CO_2_. Herein, we report a highly efficient cobalt pyridonate catalyst (TOF >12 000 h^−1^) that operates under very mild conditions with perfect chemo-specificity toward the formate reduction level. Adjustment of reaction conditions enables further reduction to the methanol level (Scheme 1, bottom).

## Results and discussion

Pyridonates confer great versatility of structure and reactivity patterns onto a metal complex by virtue of their multiple binding modes, wide stereoelectronic variation by substituents, hemilability, and redox-activity.^19^ Upon employment of polar substrates, ligand–metal cooperativity may be effectively exploited for dual activation modes at the metal and the ligand sites. From the presence of a pyridonate ligand, a strongly coordinating phosphine, and a bulky spectator ligand in the coordination sphere of a cobalt complex, we anticipated the suppression of unwanted aggregation, coordinative saturation with substrate molecules and at the same time favour a cobalt-pyridonate-centered hydroboration.^19^ Consequently, we prepared the easily accessible complex Cp*Co^II^(P∩N) (Co1) with the 6-phosphinomethyl-2-pyridonate ligand (L1).^20^ 6-Methyl-pyridone was converted to the phosphinopyridone L1H by double lithiation and substitution with ClP^*i*^Pr_2_. Base-mediated reaction of L1H with [Cp*CoCl]_2_ gave the desired half–sandwich complex Co1 in 60% yield (recrystallized from MeCN). Single crystal structure analysis of the air-sensitive complex documented the chelating *ĸ*^2^-P,N-coordination to cobalt and a pendant C=O double bond moiety with a C1–O1 bond distance of 1.243(2) Å.^21,22^ The magnetic moments (Evans method: 1.89 *μ*_B_; SQUID: T-dependent *χ*_M_T at dc field of 0.5 T, 1.90 *μ*_B_) are in full agreement with a 17-electron complex and a low-spin Co(ii) center (d^7^, *S* = ½, see Fig. S41†). Co1 is highly soluble in THF and toluene, moderately soluble in acetonitrile, sparingly soluble in ether and insoluble in hexane.^23^

We initiated our investigations into the catalytic reduction of CO_2_ with the hydroboration reaction in the presence of the inexpensive reductant pinacolborane (HBpin) and the pyridonate complex Co1.^23^ In contrast to the use of strongly hydridic boranes (*e.g.* L → BH_3_, MBH_4_, 9-BBN) that can operate in the presence of simple Lewis basic catalysts^24^ or under catalyst-free conditions,^25,26^ the use of the less reactive HBpin may enable higher selectivities and controlled access to the individual reduction intermediates (formate, acetal, methoxy levels). Consequently, the solution of catalyst and HBpin in THF-d_8_ was degassed and an ambient pressure of CO_2_ (1 bar) was applied. The reaction progress was monitored by ^1^H NMR spectroscopy (*vs.* internal mesitylene).

Full conversion was observed with 1 mol% Co1 after only 5 min exposure to 1 bar CO_2_ at room temperature (*i.e.* in the first recorded ^1^H NMR spectrum). Furthermore, the NMR spectrum exhibited perfect chemoselectivity toward the borylformate HCO_2_BPin which had formed as the only product in 100% yield (Table 1, entry 1). The same productivity was afforded with 0.1 mol% catalyst loading after 5 min at 1 bar CO_2_ (entry 2). A slightly altered reaction (0.85 mol% Co1, 2 bar CO_2_) gave full conversion after 2 min (entry 4). Change of solvents to benzene and acetonitrile and a neat reaction gave lower yields (83/21/32%, entry 5), respectively. A wide set of control reactions were performed that documented the crucial role of each component of the modular catalyst Co1: The diphenylphosphino derivative of the catalyst (Co2) afforded similarly perfect chemoselectivity toward the borylformate, but with only 34% yield (entry 6). The hydroboration did not proceed in the absence of catalyst (entry 7). The pyridone-free complexes [Cp*CoCl]_2_ and Cp*_2_Co (Co3 and Co4) were no competent catalysts, respectively (entry 8). The use of pyphos (which can be viewed as a truncated deoxo-derivative of L1) with Co3 gave very low conversion and low yield of the borylformate; whereas the *in situ* formed catalyst (from L1H, ^*t*^BuOK, and Co3) afforded good conversion (entries 9 and 10). The ligand alone showed no activity in its neutral form (L1H) or by deprotonation with potassium hexamethyldisilazide (L1K), respectively (entry 11). Likewise, conversion of L1H to the borylated derivative L1Bpin – which may be operative under hydroboration conditions - did not afford an active catalyst (entry 12). It is important to note that the catalytic hydroboration of CO_2_ operated with low amounts of the pre-catalyst Co1 (0.1 mol%) under very mild conditions (room temp., 1 bar CO_2_) in 5 min reaction time to completion and perfect chemoselectivity toward the borylformate (100% yield).

**Table 1:** Selected optimizations of the cobalt-catalyzed hydroboration of CO_2_^a^

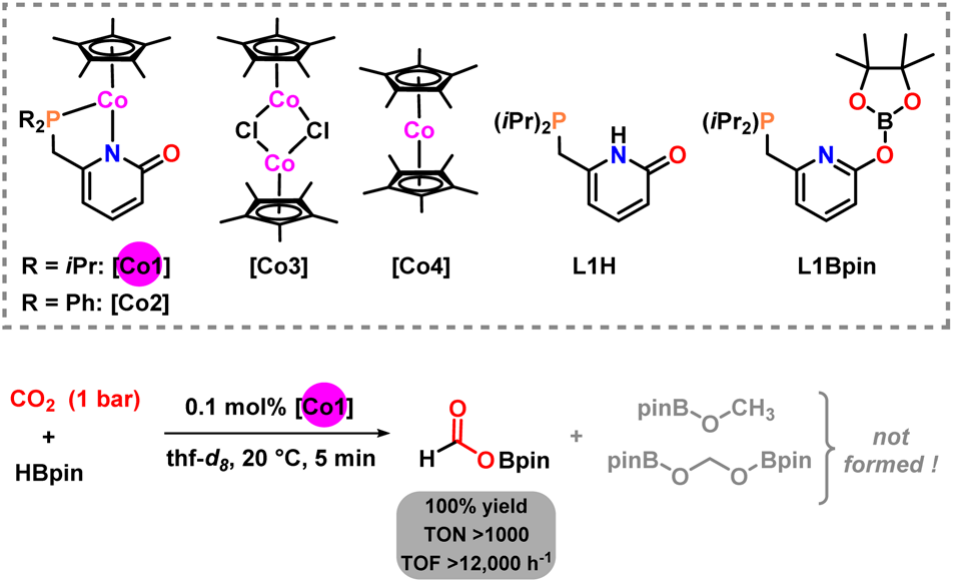		
Entry	Change from conditions above^a^	Yield [%]
1	1 mol% [Co1]	100
**2**	**None**	**100**
3	0.05 mol% [Co1]	33
4	0.85 mol% [Co1], 2 bar CO_2_, 2 min	100
5	C_6_D_6_, CD_3_CN, neat; each with 1 mol% [Co1]	83/21/32
6	1 mol% [Co2]	34
7	without catalyst	0
8	1 mol% [Co3] or [Co4]	0/0
9	2 mol% [pyphos + Co3]	8
10^b^	2 mol% [L1H + ^*t*^BuOK + Co3]	56
11	2 mol% [L1H] or [L1H + Khmds]	0/0
12^c^	2 mol% L1-Bpin	0

a Reaction conditions: an NMR tube was charged in argon-filled glovebox with HBpin (0.27 mmol), mesitylene (as internal NMR reference), the catalyst (solid or stock solution), and 0.6 mL solvent. The mixture was degassed by two cycles of freeze–pump–thaw and backfilled with 1 bar CO_2_. The NMR tube was sealed, shaken vigorously, and after 5 min the gas was released and the reaction subjected to ^1^H and ^11^B NMR analysis. ^1^H NMR yields were determined by integration *vs.* internal mesitylene as an average of two runs.

b Equimolar amounts of L1H, ^*t*^BuOK and Co3 were pre-mixed in a vial.

c A stock solution of L1Bpin in THF-d_8_ was prepared by heating equimolar amounts of L1H and HBpin at 60 °C for 16 h.

The active pre-catalyst (Co1) operated with a turnover number (TON) of greater than 1000 and a turnover frequency (TOF) of greater than 12 000 h^−1^ (determined after 5 min at full conversion; limited by time of sampling and ^1^H NMR analysis, entry 2). To the best of our knowledge, these values document a higher catalytic activity than all literature methods based on main group element and 3d transition metal catalysts. There is a single metal-catalyzed hydroboration of CO_2_ with HBpin that exhibited higher activity utilizing a Pd-silyl pincer complex (TON 37 200 after 1 day; 63 500 after 5 day; TOF 8500 h^−1^).^5^

The choice of using pinacolborane (HBpin) as reductant for highly selective hydroboration of CO_2_ was evident from a brief screening of alternative boranes (Table 2). The observed trend of borane reactivities can be partially interpreted with their thermodynamic hydridicities (Δ*G*° (H^−^).^27^ The least hydridic borane HBcat (Δ*G*° (H^−^) = 159 kcal mol^−1^) afforded very low conversions and only minor amount of reduction product (8% methoxycatecholborane). The absence of the borylformate may be a consequence of lower steric bulkiness of the catechol *vs.* the pinacol substituent. 9-BBN (more hydridic with a Δ*G*° (H^−^) = 99 kcal mol^−1^) gave full conversion in <1 h to a mixture of the diborylacetal (46%) and the methoxy borane (26%). Again, no formate intermediate was observed. The most hydridic borane in this series, BH_3_·SMe_2_ (Δ*G*° (H^−^) = 77 kcal mol^−1^), led to rapid catalyst decomposition to a dark precipitate without any detectable formation of CO_2_ reduction products.

**Table 2:** Selection of boranes and chemoselectivities of CO_2_ reduction. a

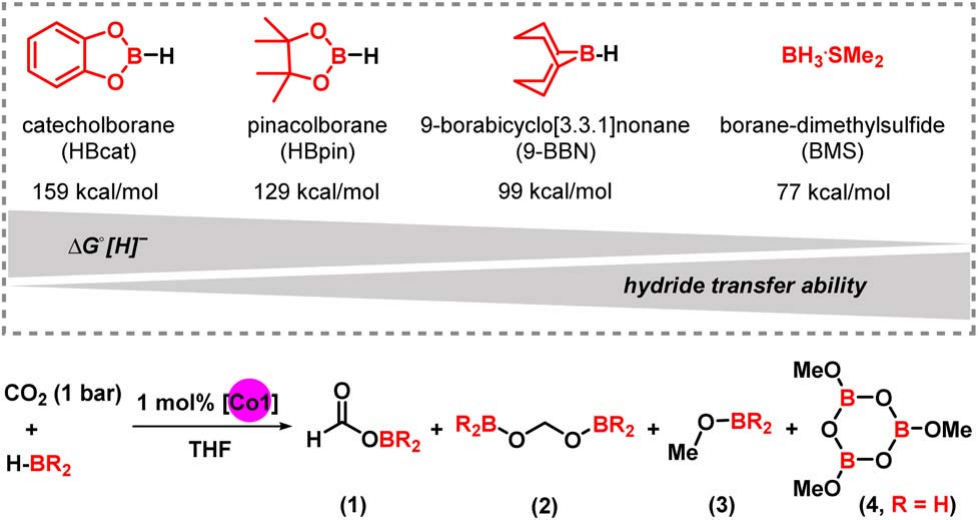				
Entry	Borane	*T* [^o^C]	*t* [h]	1/2/3/4 [%] b
1	HBcat	60	18	0/0/9/-
2	**HBpin**	20	0.1	**100**/0/0/-
3	9-BBN	20	24	0/46/26/-
4	BH_3_·SMe_2_	60	18	-/–/–/0

a Reaction conditions: an NMR tube was charged with HBpin (0.27 mmol), mesitylene (as internal NMR reference), Co1 (2.7 μmol, 1 mol%), and 0.6 mL THF-d_8_. The mixture was degassed by two cycles of freeze–pump–thaw and backfilled with 1 bar CO_2_ for 5 min. The NMR tube was sealed, shaken vigorously, (heated in an oil bath if required). Reaction progress was monitored by ^1^H and ^11^B NMR. ^1^H NMR yields were determined by integration *vs.* internal mesitylene as an average of two runs.

b 1, 2 and 3 equiv. of HBR_2_ are required for the formations of products 1, 2 and 3, respectively. 1 equiv. BH_3_·SMe_2_ and 3 equiv. CO_2_ are required to produce 4. Equimolar amounts of diboryloxide and methoxyborane 3 are formed.

**Scheme 3:**
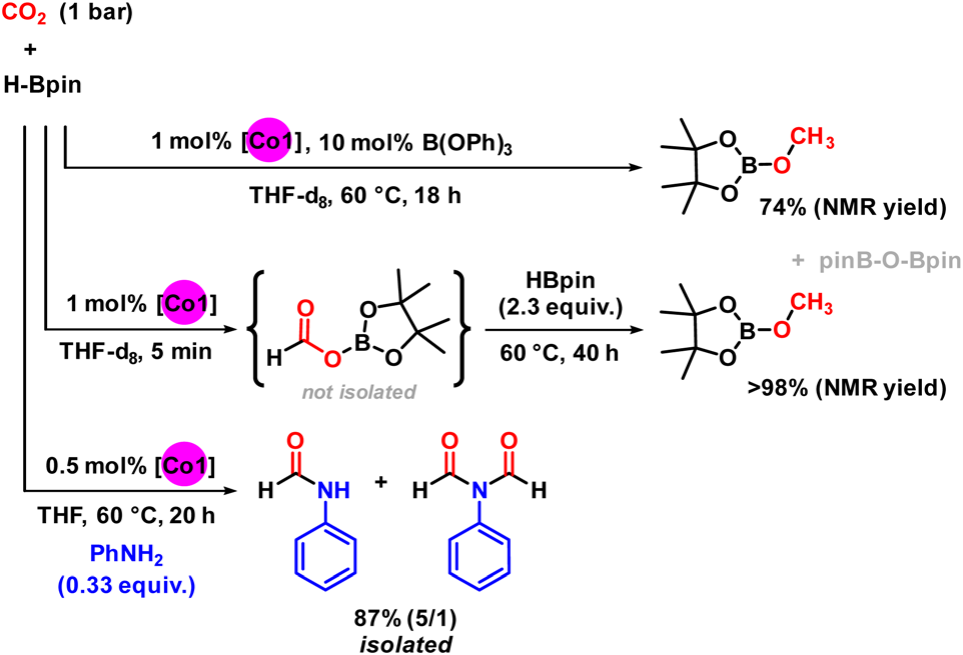
Variations of the general protocol. Top: selective hydroboration to the boryl-formate and methoxyborane. Bottom: *in situ* formylation to give *N*-phenyl formamides.

While the hydroboration of CO_2_ with HBpin and catalytic Co1 under standard conditions (1 bar CO_2_, THF, 20 °C, 5 min) cleanly afforded the boryl formate in perfect yield and selectivity, change of the reaction conditions enabled onward reduction to the methanol level. Addition of the Lewis acid B(OPh)_3_ as co-catalyst (10 mol%) fully inhibited the hydroboration at 20 °C (no conversion of HBpin after 1 h), but afforded the corresponding methoxyborane as single reduction product in 74% yield after 16 h at 60 °C (Scheme 3, top).^5,14^ More conveniently, full conversion to the methoxyborane could be easily achieved when adding excess amounts of HBPin to the crude boryl formate and further reaction at 60 °C (Scheme 3, middle, and Fig. S1 and S2†). The synthetic utility of the borylformate product was explored by addition of aniline to the hydroboration reaction (Scheme 3, bottom). Reaction of aniline and 3 equiv. HBpin in the presence of Co1 (1 mol%) in THF under 1 bar CO_2_ at room temperature resulted in the formation of a mixture of HCO_2_Bpin and the undesired dehydro-coupling product PhNHBpin (2/1). The same reaction with 0.5 mol% Co1 at 60 °C gave clean formylation of the aniline to afford *N*-formanilide and *N*,*N*-diformyl aniline in overall 87% isolated yield (5/1; see Fig. S8† for details).^12^

### Mechanistic studies

**Scheme 4:**
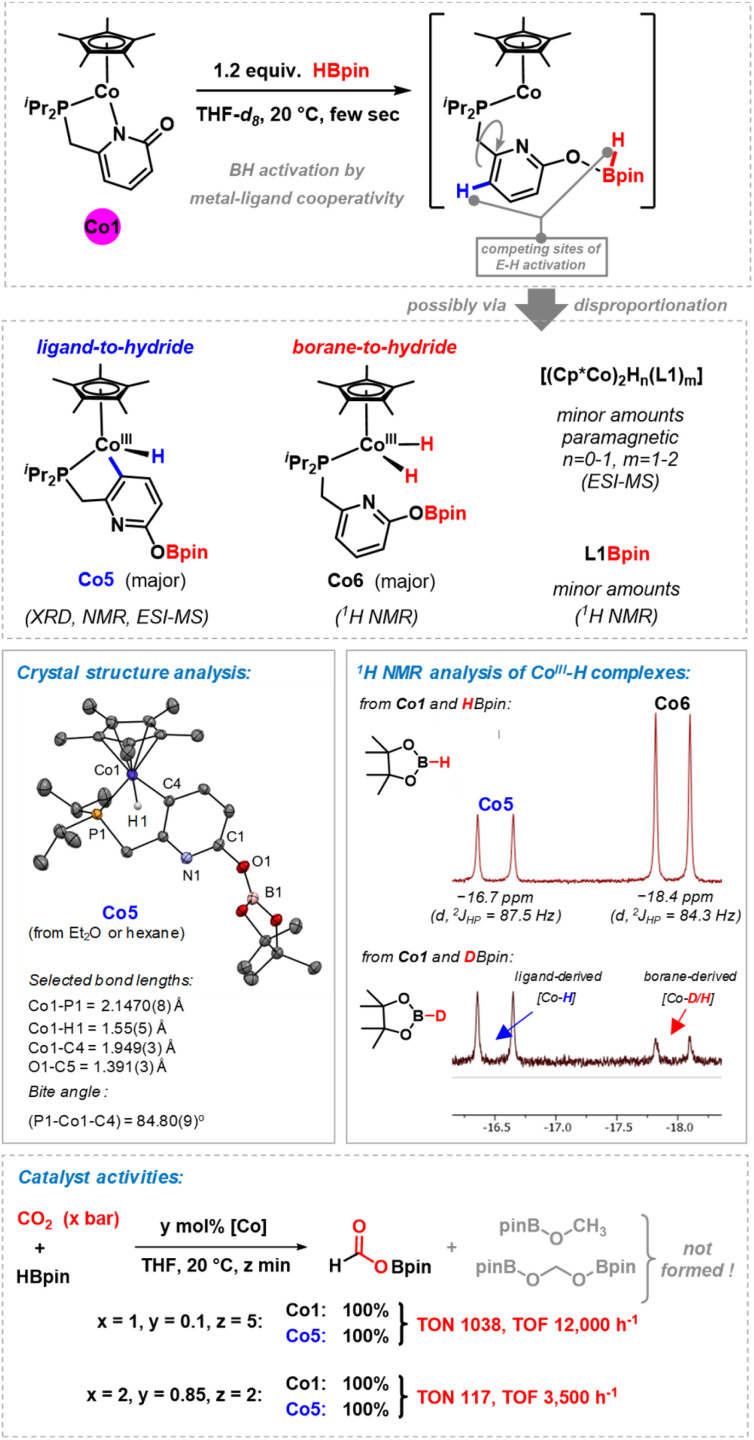
Selected mechanistic studies. Top: synthesis and characterization of potential catalyst intermediates, including the isolation and characterization of key cobalt hydride complexes Co5 and Co6.^23^ Bottom: equal catalytic activities of precatalyst Co1 and proposed catalyst species Co5 under different conditions.

A set of preparative and spectroscopic experiments were performed in order to gain insight into the nature of the catalytically active species (Scheme 4). Similar reaction conditions and catalysts have been applied to the regioselective hydroboration of pyridines, which may operate by a related mechanism.^23^ Stoichiometric reaction of the cobalt–pyridonate complex Co1 with pinacolborane (1.2 equiv.) in THF-d_8_ resulted in immediate color change from orange to dark green or even blackish. We postulate the formation of a transient labile cobalt(ii) hydride complexes which underwent rapid disproportionation,^28^ possibly *via* dinuclear hydride or pyridonate-bridged species. ^1^H NMR monitoring indicated the formation of diamagnetic (and minor amounts of paramagnetic) species: (i) very minor amounts of the borylated ligand L1Bpin were observed by ^1^H NMR. (ii) We postulate the formation of dinuclear (or higher) complexes of the formula [(Cp*Co)_2_H_*n*_(L1)_*m*_] as minor paramagnetic species with Co(i), Co(ii), or mixed valence states. ESI-MS spectra showed *m*/*z* = 612.2216 (*n* = 0, *m* = 1) and 837.3498 (*n* = 1, *m* = 2). Similar hydride- and pyridonate-bridged dinuclear complexes were prepared from [Cp*CoCl]_2_/LiAlH_4_ (ref. 29) and from [Cp*IrCl(2-pyridonate)]^30^ by hydrogen transfer, respectively. (iii) Most interestingly, two distinct cobalt hydride complexes were formed, which differ in the origin of their hydride ligands (Scheme 4, middle and Fig. S13 and S14†).^23^ The diamagnetic monohydridocobalt(iii) complex Co5 (−16.5 ppm; ^2^*J*_(P,H)_ = 87.3 Hz) formed by C–H activation at the pyridine ligand moiety. The dihydrido phosphine cobalt(iii) complex Co6 exhibited a more upfield hydride signal (−18.0 ppm; ^2^*J*_(P,H)_ = 84.4 Hz), which is in agreement with closely related CpCo^III^H(PR_3_) complexes.^31–33^ The origin of the hydride ligands was furnished by the same reaction of Co1, but with DBpin instead of HBpin: Similar intensity of the ^1^H resonance of Co5 (as from HBpin) was observed but very low intensity of the borane-derived hydride signal of Co6 (Fig. S15†). Complex Co6 also formed by addition of HBpin to a solution of Co5, so that Co6 can be viewed as an overreduction product of Co5. A dinuclear derivative of Co6 could be isolated by crystallization.^23^ Complex Co5 was crystallized from diethylether and *n*-hexane (20% crystal yield). The single crystal structure analysis showed a three-legged piano stool complex with terminal hydride and a 5-membered metallocycle with P∩C-coordination of the pyridonate ligand. The 2-oxo position of the ligand bears a Bpin substituent, which supports the notion of a dual activation mode in this hydroboration reaction by the cobalt center and the basic oxygen site.^23^

**Scheme 5:**
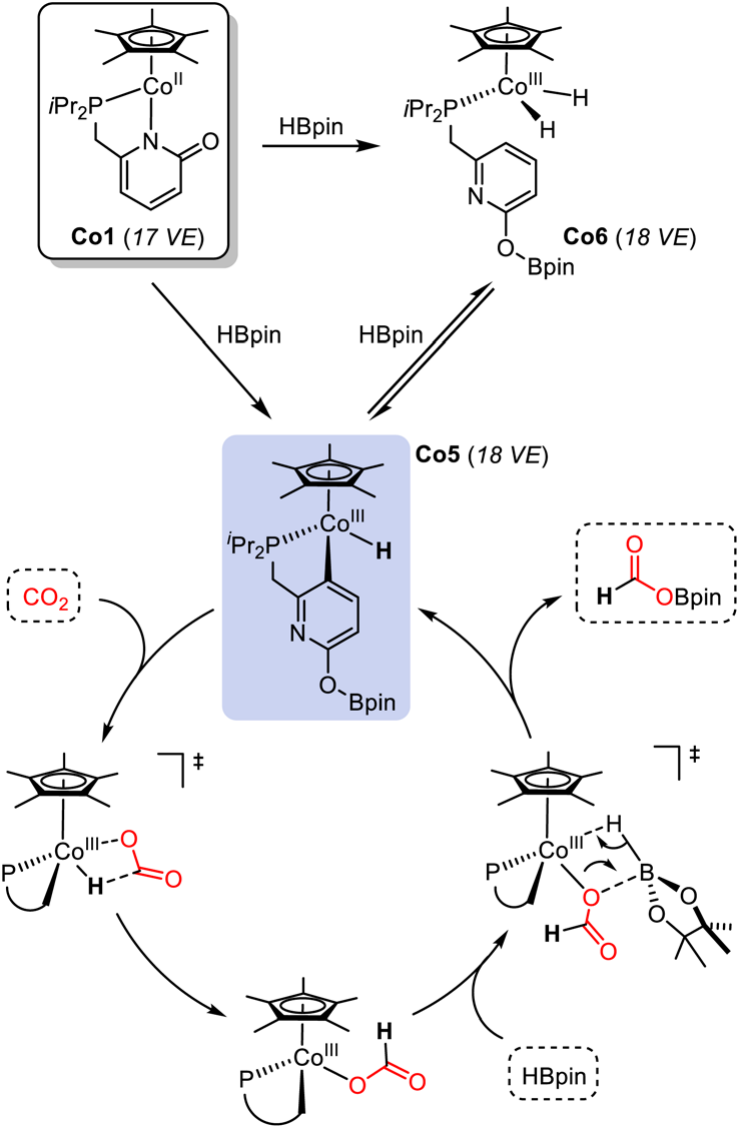
Postulated reaction mechanism *via*Co5 as key catalytic intermediate. A closely related mechanism was postulated for a cobalt-catalyzed hydroboration of pyridines.^23^

In the catalytic hydroboration of CO_2_, complex Co5 was equally active as pre-catalyst Co1 under the standard conditions, which may indicate the role of Co5 as catalytically active species (Scheme 4, bottom). Full conversion of HBpin to the borylformate was observed after reaction at room temperature for 5 min with 1 mol% Co5. Co5 is stable toward higher excess amounts of borane. Addition of up to 4 equiv. HBpin did not result in any detectable shift or disappearance of the ^1^H NMR resonance at – 16.5 ppm. Further evidence that Co5 is a competent catalyst was derived from its instantaneous reaction with CO_2_ which was followed by ^1^H NMR spectroscopy. The ^1^H signal of the Co5 hydride ligand at −16.5 ppm disappeared instantaneously while the −18.0 ppm hydride resonance of Co6 remained unchanged for several hours before it only slowly disappeared overnight. We therefore postulate a reaction mechanism that involves rapid conversion of Co1 into Co5. This key catalytic cobalt hydride intermediate directly transfers a hydride onto CO_2_. Borane activation may proceed *via* a σ-bond metathesis event that releases the formyl borate HCO_2_Bpin and regenerates the active catalyst Co5 (Scheme 5).^23^

## Conclusions

The hydroboration of CO_2_ was realized under very mild conditions (1 bar CO_2_, 20 °C, 5 min) with low catalyst loading (0.1 mol%) of a simple cobalt catalyst. Perfect chemoselectivity toward the borylformate (100% yield) and very high catalyst activity (TON 1000, TOF 12 000 h^−1^) were observed. Mechanistic studies revealed two distinct modes of hydride complex formation under the reaction conditions: ligand-centered CH activation to the monohydride cobalt(iii) complex Co5 and borane-induced formation of the dihydrido-cobalt(iii) complex Co6. The modular composition of Co1 and identification of Co5 as active catalyst will prompt further studies into the generation of active metal hydride complexes. This concept has already proven successful in a new pyridine reduction method.^23^ Further applications to the wide space of hydrofunctionalization and hydrogenation reactions are easily foreseen.

## Data availability

The data supporting this article have been included as part of the ESI.†

## Author contributions

A. F.: conceptualization, investigation, validation, writing draft. L. L.: investigation, validation. A. J. v. W.: conceptualization, draft review and edit, visualization, supervision, administration, funding acquisition.

## Conflicts of interest

There are no conflicts to declare.

## Supplementary Material

RA-015-D5RA01656H-s001
