# Supplementary material for: Chemospecific cobalt-catalyzed hydroboration of CO2
Source: RSC Adv. 2025 Jun 3;15(23):18512–7. doi: 10.1039/d5ra01656h (PMC12131809; doi:10.1039/d5ra01656h)
Supplement: RA-015-D5RA01656H-s001 [file RA-015-D5RA01656H-s001.pdf]

Supplementary Information (SI) for RSC Advances.
This journal is © The Royal Society of Chemistry 2025

# Supplementary Information

## Chemospecific Cobalt-Catalyzed Hydroboration of $CO_2$

Andrey Fedulin,[a,b] Lea Luxenberger,[a] and Axel Jacobi von Wangelin[a]*

[a] Dept. of Chemistry, University of Hamburg, Martin Luther King Platz 6, 20146 Hamburg, Germany. E-mail: axel.jacobi@uni-hamburg.de

[b] Dept. of Chemistry, University of Regensburg, 93040 Regensburg, Germany.

## General Information

All experiments for air-sensitive compounds were performed under an atmosphere of dry argon, by using the standard Schlenk and glove box techniques.

*Chemicals and solvents:* Solvents ($Et_2O$, *n*-hexane, toluene, acetonitrile) were purified by an SPS solvent purification system under $N_2$; THF was distilled over sodium and benzophenone. Dry solvents were stored over molecular sieves (4 Å). Deuterated solvents (THF-$d_8$, $C_6D_6$, toluene-$d_8$, acetonitrile-$d_3$) were distilled over sodium or potassium metal and stored over molecular sieves (4 Å).

*Ligand and complex syntheses:* Pentamethylcyclopentadiene was synthesized according to a literature procedure.[3] Triphenylborate was purchased from *Sigma Aldrich* in 97% purity. Aniline was obtained from *Th. Geyer*, distilled in vacuum and dried over molecular sieves (4 Å) prior to use to gain >99% purity. Chlorodiphenylphosphine (98%) was purchased from *BLD pharm;* chlorodiisopropyl-phosphine (96%) from *Fisher Scientific.* The syntheses of 2-(2-(diphenylphosphanyl)-ethyl)pyridine (PyPhos),[1] 6-((diphenylphosphanyl)methyl)pyridin-2(1H)-one,[2] and the cobalt complexes decamethylcobaltocene $(Cp^*CoCl)_2$[3] and **Co2**[4] followed previously reported literature procedures.

*Catalysis:* Pinacolborane (HBpin) from *Sigma Aldrich* (97%) was distilled or directly used and stored in the freezer at -37°C under argon. Catecholborane (HBcat) was purchased from *VWR* (97%). 9-Borabicyclo[3.3.1]nonane (9-BBN, 0.5 M in THF) and borane dimethylsulfide (BMS) were both purchased from *Sigma Aldrich*, then stored and handled under argon. 6-Methyl-2-pyridone (98%) was purchased from *BLD Pharm* and sublimed in vacuum prior to use. Carbon dioxide gas (99.999 vol%) was purchased from Westfalen AG.

*NMR spectroscopy* ($^1H$, $^{13}C$, $^{11}B$, $^{31}P$). Nuclear magnetic resonance spectra were recorded on a Bruker Avance 400 (400 MHz), Bruker Avance 500 (400 MHz) and Bruker Avance 600 (600 MHz). $^1H$-NMR: The following abbreviations are used to indicate multiplicities: s = singlet; d = doublet; t = triplet, q = quartet; m = multiplet, dd =doublet of doublet, dt = doublet of triplet, dq = doublet of quartet, ddt = doublet of doublet of quartet. Chemical shifts δ are given in ppm referenced to the residual solvent peak.

*Solution magnetic moment* was determined by performing an NMR experiment following the procedure of Evans[5].

*Melting points* of the compounds were measured on a *DigiMelt SRS* instrument using a glass capillary sealed under vacuum.

*Elemental analyses* were determined in an inert atmosphere by the analytical service facility at the Dept of Chemistry of the University of Hamburg.

UV/visible absorption spectra were recorded at room temp. on an Agilent Cary 5000 UV-Vis-NIR double beam spectrometer in a 10 mm quartz cuvette with a Teflon valve.

*FT-IR spectra* were recorded on an Agilent Cary 630 FTIR with ATR at 20 °C inside a nitrogen-filled glovebox.

*ESI-MS* was measured on a Q-TOF mass spectrometer micrO-TOF-Q II (Bruker Daltonik) with ESI, APCI, and cryospray-ionization sources (Dept. of Chemistry, University of Göttingen).

## Synthetic Procedures

*Synthesis of 6-((diisopropylphosphanyl)methyl)pyridin-2(1H)-one (L1H)*

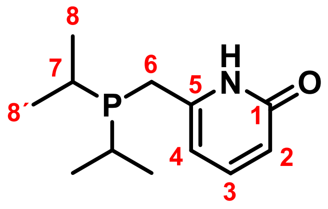

A suspension of 6-methyl-2-pyridone (1.50 g, 13.8 mmol, 1.00 equiv.) in THF (15 mL) was cooled to 0 °C and *n*-butyl lithium (1.6 M in hexane, 18 mL, 29 mmol, 2.1 eq.) was added dropwise over a period of 10 min while stirring. During the addition, the reaction mixture turned orange and the colourless solid dissolved continuously. The orange solution was then stirred at 0 °C for 2 h. Subsequently, the reaction mixture was cooled to -78 °C and added dropwise to a solution of chlorodiisopropylphosphine (2.20 mL, 13.8 mmol, 1.00 eq.,) in THF (10 mL) at -78 °C over 20 min with vigorous stirring. After stirring the orange reaction mixture at -78 °C for 1 h, it was allowed to warm up to room temperature and stirred for an additional 19 h. The solvent was removed under reduced pressure and the degassed aqueous $NH_4Cl$ solution (3.3 M, 30 mL) was added to the red residue. The suspension was stirred for 10 min and then extracted with DCM (4x15 mL). The combined organic layers were dried over anhydrous $MgSO_4$, filtered and the solvent was evaporated *in vacuo*. The resulting yellow solid was crystallized from hexane at -30 °C. Slightly yellow crystalline solid was collected by filtration, washed with cold hexane and dried in vacuum. Yield: 2.13 g (69%).

**m.p.** = 84–85 °C.

**$^{1}$H-NMR** (500.1 MHz, $C_6D_6$, 24.9 °C): ™ [ppm] = 14.34 (s, 1 H, -NH), 6.85 (dd, $^{3}J_{(H,H)}$ = 9.1 Hz, $^{3}J_{(H,H)}$ = 7.0 Hz, 1 H, 3-H), 6.42 (d, $^{3}J_{(H,H)}$ = 9.1 Hz, 1 H, 2-H), 6.09–6.06 (m, 1 H, 4-H), 2.69 (s, 2 H, 6-H), 1.61 (heptd,f $^{3}J_{(H,H)}$ = 7.1 Hz, $^{2}J_{(P,H)}$ = 1.1 Hz, 2 H, 7-H), 1.01–0.93 (m, 12 H, 8-H and 8'-H).**$^{13}$C NMR** (125.8 MHz, $C_6D_6$, 24.7 °C): ™[ppm] = 166.4 (C-1), 149.9 (d, $^{2}J_{(P,C)}$ = 11.3 Hz, C-5), 141.5 (C-3), 116.4 (d, $^{5}J_{(P,C)}$ = 1.7 Hz, C-2), 106.1 (d, $^{3}J_{(P,C)}$ = 11.1 Hz, C-4), 26.9 (d, $^{1}J_{(P,C)}$ = 24.7 Hz, C-6), 23.9 (d, $^{1}J_{(P,C)}$ = 15.8 Hz, 2 C, C-7), 19.7 (d, $^{2}J_{(P,C)}$ = 15.0 Hz, 2 C, C-8/8'), 19.1 (d, $^{2}J_{(P,C)}$ = 11.3 Hz, 2 C, C-8/8'). **$^{31}$P{$^{1}$H}-NMR** (202.5 MHz, $C_6D_6$, 25.1 °C): ™ [ppm] = 25.6 (s).

**Elemental analysis**: calc. for $C_{12}H_{20}NOP$: C 63.98, H 8.95, N 6.22; found: C 63.72, H 8.90, N 6.23.

### *Synthesis of complex **Co1** [6]*

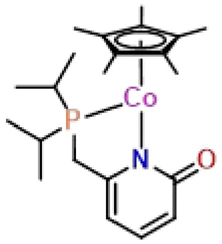


A solution of potassium *tert*-butoxide (458 mg, 3.96 mmol) in 10 mL THF was added dropwise to the solution of the L1H (850 mg, 3.77 mmol) in 120 mL THF. The reaction mixture was stirred at ambient temperature for 2 hours followed by dropwise addition of the solution of $[Cp^*CoCl]_2$ (866 mg, 3.77 mmol) in 10 mL THF. The mixture turned from slightly yellow to dark orange (almost black). After stirring at ambient temperature overnight, all volatiles were removed under vacuum, orange solid residue was extracted with toluene (30 mL) and the toluene extract was filtered through a short pad of Celite®. The filtrate was concentrated in vacuum and the brown-orange solid residue was crystallized from 70 mL acetonitrile at -30 °C. A large crop of dark-red crystals was collected by filtration, washed with hexane and dried in vacuum. Yield: 954 mg (60%). Single crystal suitable for X-ray analysis was collected during the described crystallization procedure.

**m.p.** = 184–186 °C.

**Evans-NMR** (300.2 MHz, $C_6D_6$, 24.9 °C): $\mu = 1.89\ \mu_B$, unpaired electrons (spin-only) $n = 1$.

**UV-Vis** (THF, 0.05 mg/mL): $\lambda_{max}$ ($\varepsilon$) = 396 nm (3358 $L{\cdot}mol^{-1}{\cdot}cm^{-1}$), 332 nm (8633 $L{\cdot}mol^{-1}{\cdot}cm^{-1}$), 240 nm (15906 $L{\cdot}mol^{-1}{\cdot}cm^{-1}$), 209 nm (18409 $L{\cdot}mol^{-1}{\cdot}cm^{-1}$).

**Elemental analysis**: calc. (%) for $C_{22}H_{34}CoNOP$: C 63.15, H 8.19, N 3.35; found: C 62.87, H 8.28, N 3.28.

**ESI-MS:** ($C_{22}H_{34}CoNOP$) Calculated: 418.1704; Found: 418.1649.

*Synthesis of complex* ***Co5*** *[6]*

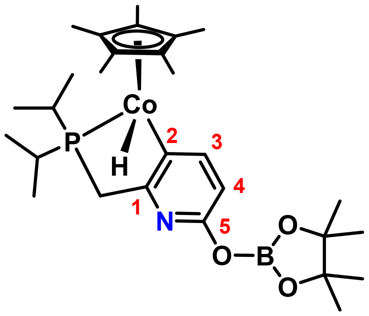


Complex **Co1** (157 mg, 0.375 mmol) was dissolved in 3.0 mL THF and HBpin (67 µL, 0.462 mmol, 1.2 equiv.) was added. Colour change from orange-red to black was observed. Reaction mixture was stirred at room temperature for 22 h. All volatiles were removed in vacuum and the oily residue was treated with approx. 1 mL hexane followed by solvent removal in vacuum (manipulation was repeated two times). Addition of the new portion of hexane (approx. 2 mL) was accompanied by formation of the orange solid. Solid was collected by filtration, extracted with approx. 5 mL hexane and residual solid was dissolved in 2.0 mL $Et_2O$. Both hexane and $Et_2O$ fractions were stored overnight at -35°C. From hexane fraction dark-orange crystals (27 mg) were isolated. Diethyl ether fraction yielded orange microcrystalline solid (27 mg). Single crystals suitable for X-ray analysis were obtained by slow evaporation of the hexane fraction. Both solids, collected from hexane and $Et_2O$ were proved to be identical Co-H species on the basis of $^1$H NMR analysis. Combined yield 26%.

**$^1$H NMR** (600 MHz, THF-$d_8$) δ [ppm] = 7.53 (d, $^3J_{H,H}$ = 8.1 Hz, 1 H, H-3), 6.34 (d, $J$ = 8.3 Hz, 1H, H-4), 2.64 – 2.52 (m, 2H, C$\underline{H}_2$-P), 2.16 ( hept, $J$ = 7.3 Hz, 2H, *i*-Pr-C$\underline{H}$), 1.82 (s, 15H, Cp*-C$\underline{H}_3$), 1.26 (dd, 3H, dd, overlapped, 3H, *i*-Pr-C$\underline{H}_3$), 1.23 (s, 12H, Bpin-C$\underline{H}_3$), 1.17 (dd, $^3J_{P,H}$ = 12.4 Hz, $^3J_{H,H}$ = 6.8 Hz, 3H, *i*-Pr-C$\underline{H}_3$), 1.05 (dd, $^3J_{P,H}$ = 13.9 Hz, $^3J_{H,H}$ = 7.1 Hz, 3H, *i*-Pr-C$\underline{H}_3$), 0.93 (dd, $^3J_{P,H}$ = 12.9, $^3J_{H,H}$ = 6.9 Hz, 3H, *i*-Pr-C$\underline{H}_3$), -16.70 (d, $^2J_{P,H}$ = 87.5 Hz, 1H, Co-$\underline{H}$). **$^{13}$C NMR** (151 MHz, THF-$d_8$) δ [ppm] = 165.32 (d, $^2J_{P,C}$ = 19.7 Hz, C1), 157.18 (C5), 150.03 (C4), 148.50 (C2), 110.14 (C3), 93.05 (Cp*-C), 82.92 (C-Bpin), 37.15 (d, $^1J_{C,P}$ = 39.0 Hz, $\underline{C}H_2$Py), 24.93 (Bpin-$\underline{C}H_3$ overlaped with THF-$d_8$), 27.34 (d, 13.4 Hz *i*-Pr-$\underline{C}$H), 23.90 (d, $J$ = 26.1 Hz, *i*-Pr-CH), 20.07 (*i*-Pr-$\underline{C}H_3$), 19.12 (*i*-Pr-$\underline{C}H_3$), 18.78 (*i*-Pr-$\underline{C}H_3$), 18.57 (*i*-Pr-$CH_3$), 11.41 (Cp*-$\underline{C}H_3$). **$^{31}$P NMR** (243 MHz, THF-$d_8$) δ 91.63 (br). **$^{11}$B NMR** (193 MHz, THF-$d_8$) δ 21.98. **IR-FTIR** (ATR, cm$^{-1}$): $\nu_{Co\text{-}H}$ =1924.

**Elemental analysis** calculated for $C_{28}H_{46}BCoNO_3P$ C 61.66, H 8.50, N 2.57; found 61.71, 8.67, 2.57
**ESI-MS**: ($C_{28}H_{46}BCoNO_3P$) Calculated: 545.2640; Found: 545.2546.

## Catalytic $CO_2$ hydroboration

*General procedure for $CO_2$ hydroboration to formyloxyborane*

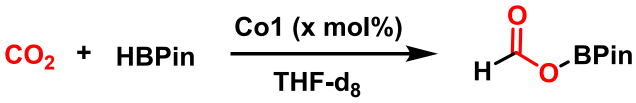

**<u>Method A</u>:** Catalyst **Co1** (1.1 mg, 2.6 µmol, 1 mol%, or selected amount of solid or aliquot of the stock solution in THF-$d_8$), internal standard mesitylene (15 µL, 0.1078 mmol) and 0.6 mL THF-$d_8$ were mixed in a scintillation vial. Pinacolborane **HBpin (41 µL, 0.27 mmol)** was added and reaction mixture was transferred to J. Young NMR tube. Solution was degassed with two freeze-pump-thaw cycles. Then solution was immersed in a liquid nitrogen bath for the third time and vacuum was applied to a frozen solution. NMR tube was lifted form the liquid nitrogen and reaction mixture was exposed to 1 bar $CO_2$. The J. Young tube was sealed, shaken vigorously and after ~5 min analyzed by $^1$H and $^{11}$B NMR analysis. $^1$H NMR yield was determined by integration of signals of formyloxyborane with reference to the internal standard mesitylene as an average of two runs.

A neat reaction was performed in HBpin as solvent (100 µL). See table 1, entry 5 in the manuscript.

For the hydroborations inside J. Young NMR tubes, the following amount of $CO_2$ was present:

**Calculated amount** of $CO_2$ $n = \frac{pV}{RT}$

J. Young NMR tube: ~1.1 bar: n($CO_2$) ≈ 0.115 mol (volume of $CO_2$ ≈ 2.45 mL)

Activity and productivity of a 0.1 mol% [**Co1**] reaction at 20°C after 5 min with full conversion to product (by $^1$H NMR):

**TON** $\frac{n(product)\ 0.27\ mmol}{n(catalyst) 0.00026\ mmol} \approx 1038$

**TOF** $\frac{TON}{t = 0.083333\ h} \approx 12000\ h^{-1}$

The TON and TOF are minimal values due to the fast rate of the reaction and the determination from datapoints of full conversion.

NMR data of $HCO_2Bpin$:[7]

**$^1$H NMR** (400 MHz, THF-$d_8$) δ [ppm] = 8.40 (s, 1H, <u>H</u>$CO_2$Bpin), 1.31 (s, 12H, $HCO_2$B<u>pin</u>). **$^{13}$C NMR** (101 MHz, THF-$d_8$) δ [ppm] = 158.54, 85.33, 24.99. **$^{11}$B NMR** (128 MHz, THF-$d_8$) δ [ppm] = 22.68.

*Sequential hydroboration of $CO_2$ and in situ formed $HCO_2Bpin$*

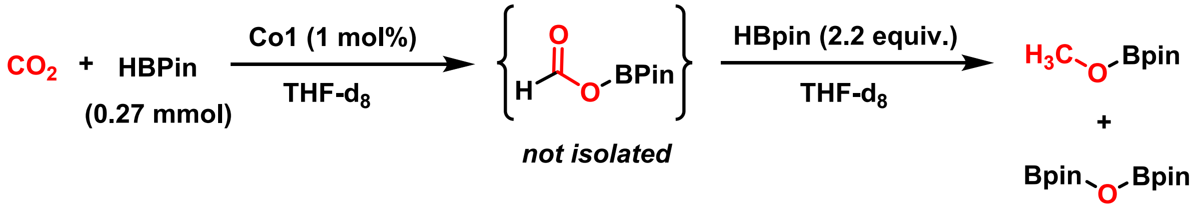


**Co1** (1.1 mg, 2.6 μmol, 1mol%), internal standard mesitylene (15 μL, 0.1078 mmol) and 0.6 mL THF-$d_8$ were mixed in a scintillation vial. Pinacolborane HBpin (41 μL, 0.27 mmol) was added and reaction mixture was transferred to J. Young NMR tube. This solution was degassed with two freeze-pump-thaw cycles. Then, the solution was immersed into a liquid nitrogen bath for the third time and vacuum was applied to the frozen solution. The NMR tube was lifted form the liquid nitrogen and reaction mixture was exposed to 1 bar $CO_2$ for 5 min. The J. Young tube was then sealed, shaken vigorously and subjected to $^1$H NMR to confirm full conversion to $HCO_2Bpin$. Then the reaction mixture was carefully degassed under static vacuum to remove $CO_2$. The J. Young tube was transferred to argon-filled glovebox and HBpin (90 μL, 0.62 mmol) was added. The J. Young NMR tube was placed in a 60 °C oil bath and reaction progress was monitored by $^1$H NMR. Colour of the reaction mixture changed gradually from yellow to bright green. After heating for 40 h, almost full conversion of $HCO_2Bpin$ to $CH_3OBpin$ and $(pinB)_2O$ was achieved.

NMR data for $CH_3OBpin$:[7,8]

**$^1$H NMR** (400 MHz, THF-$d_8$) δ [ppm] = 3.50 (s, 3H), 1.20 (s, 12H). **$^{13}$C NMR** (101 MHz, THF-$d_8$) δ [ppm] = 83.21, 52.66, 25.17. **$^{11}$B NMR** (128 MHz, THF-$d_8$) δ [ppm] = 22.35.

NMR data for $(pinB)_2O$:[7]

**$^1$H NMR** (400 MHz, THF-$d_8$) δ [ppm] = 1.21 (s, 24H) **$^{13}$C NMR** (101 MHz, THF-$d_8$) δ [ppm] = 83.52, 25.10. **$^{11}$B NMR** (128 MHz, THF-$d_8$) δ [ppm] = 21.24.

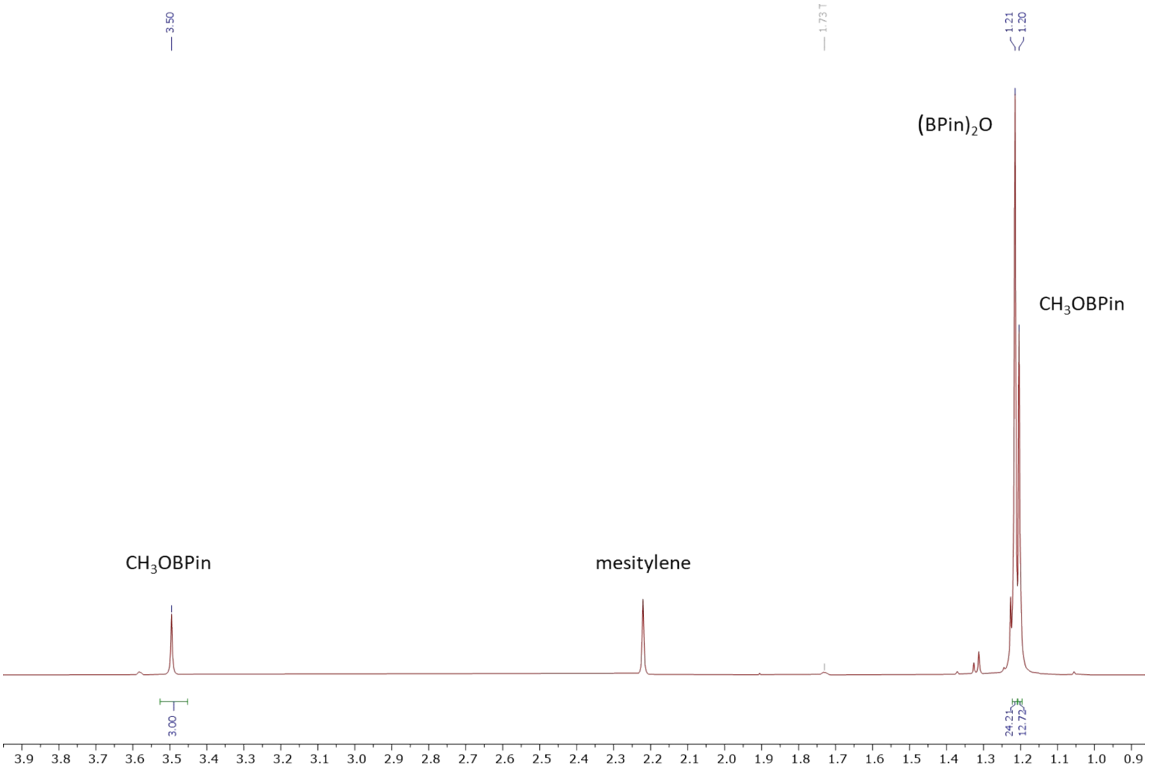


**Figure S1.** Crude $^{1}H$ NMR spectrum (400 MHz, THF-$d_8$) of $HCO_2Bpin$ hydroboration to $CH_3OBpin$.

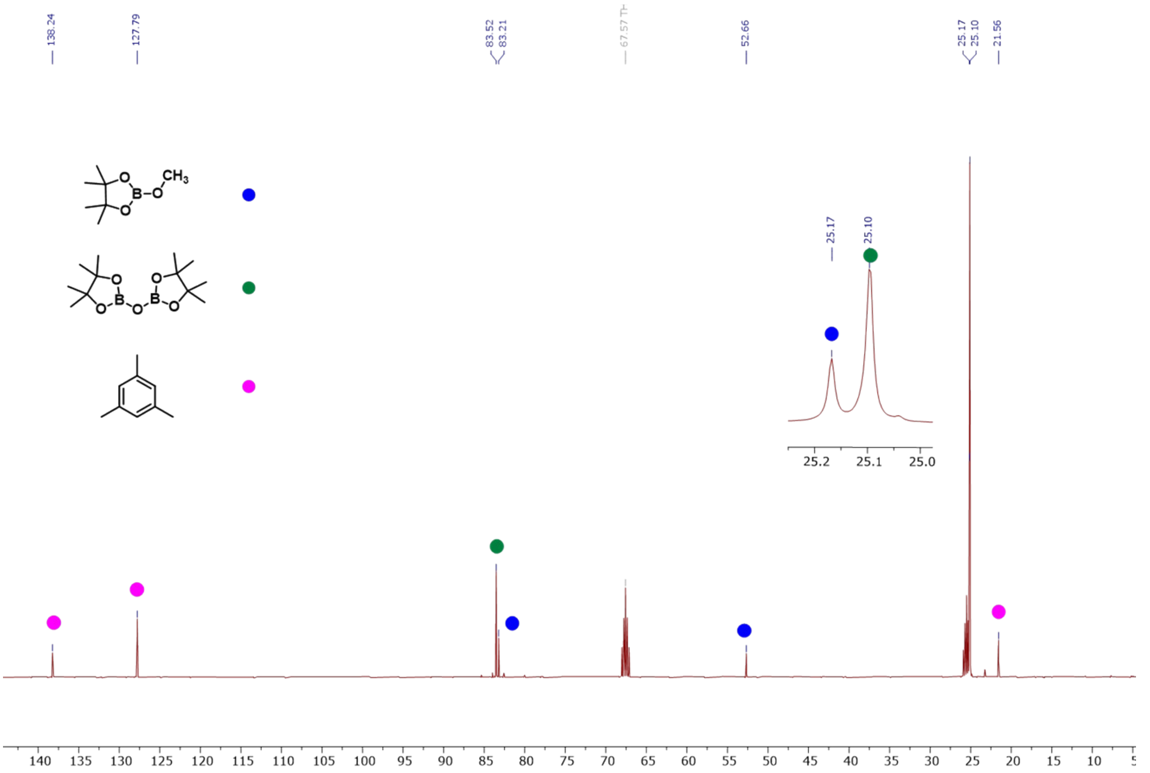


**Figure S2.** Crude $^{13}C$ NMR spectrum of $HCO_2Bpin$ hydroboration to $CH_3OBpin$.

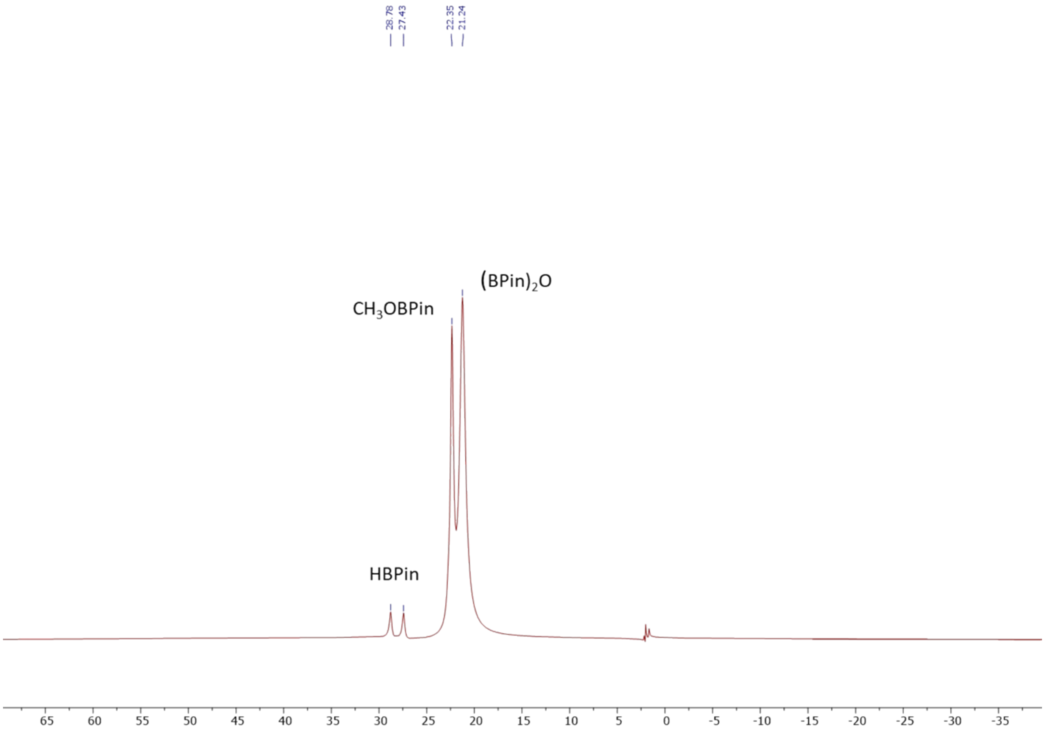


**Figure S3.** Crude $^{11}$B NMR spectrum of $HCO_2$Bpin hydroboration to $CH_3$OBpin.

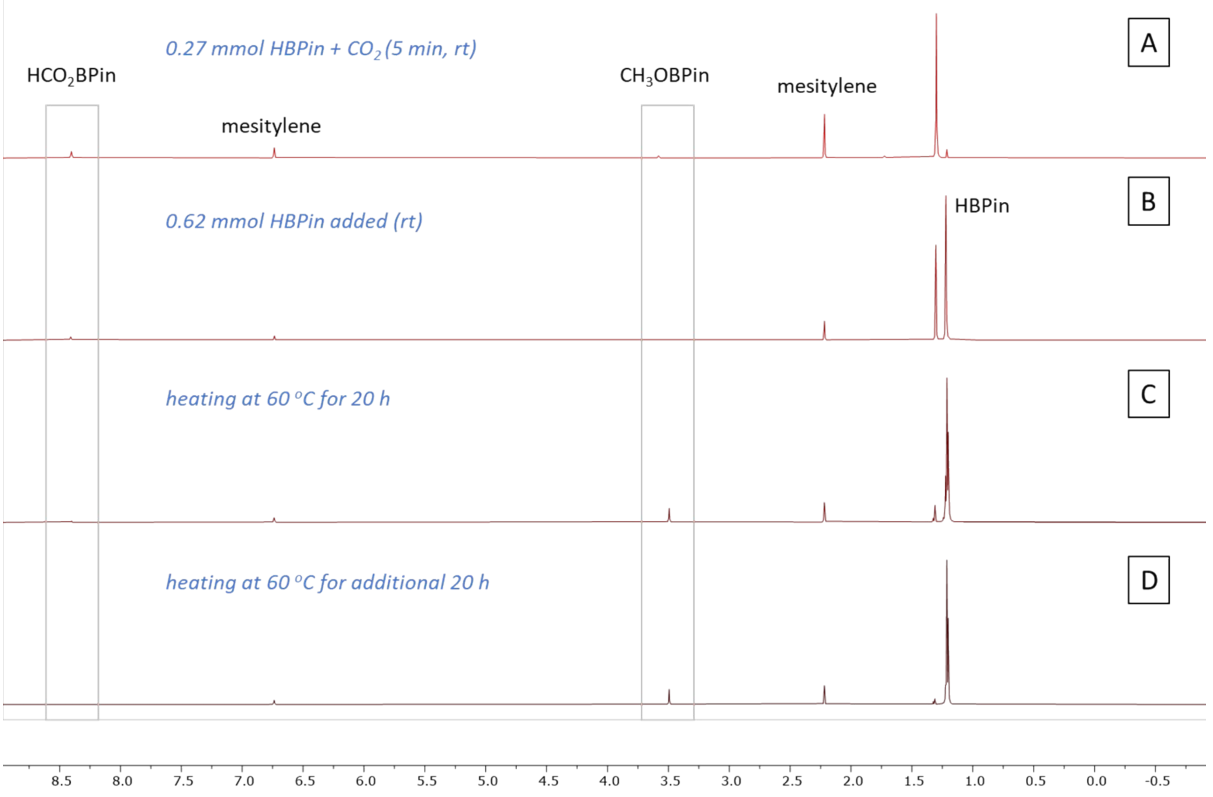


**Figure S4.** Stepwise $^{1}$H NMR monitoring of crude **Co1**-catalyzed reaction to $HCO_2$Bpin and $CH_3$OBpin **A**: HBpin (0.27 mmol) + $CO_2$, 5 min at r.t.; **B**: addition of 2.3 equiv. HBpin to reaction mixture A at rt; **C**: heating for 20 h at 60 °C, 84% $CH_3$OBpin; **D**: heating for additional 20 h at 60 °C, >99% $CH_3$OBpin.

*Lewis acid assisted hydroboration of $CO_2$*

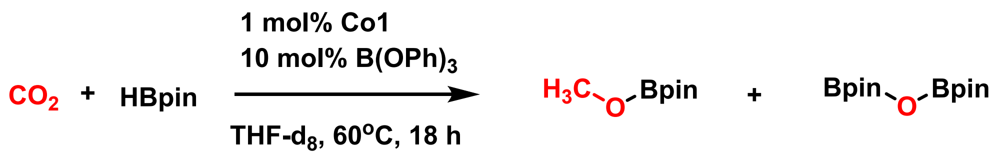


**Co1** (1.1 mg, 2.7 µmol, 1mol%), internal standard mesitylene (15 µl, 0.1078 mmol), triphenylborate $B(OPh)_3$ (7.8 mg, 0.027 mmol) and 0.6 mL THF-$d_8$ were mixed in a scintillation vial. Pinacolborane HBpin (41 µL, 0.27 mmol) was added and reaction mixture was transferred to a J. Young NMR tube. The solution was degassed with two freeze-pump-thaw cycles, then immersed into a liquid nitrogen bath for the third time and vacuum was applied to the frozen solution. The J. Young NMR tube was lifted form the liquid nitrogen and reaction mixture was exposed to 1 bar $CO_2$ for 5 min. The J. Young tube was then sealed, shaken vigorously and placed to the 60 °C oil bath. Reacton progress was monitored by $^1H$ NMR. Colour of the reaction mixture changed gradually from yellow to bright green. After heating for 18 h, 74% (NMR) of $CH_3OBpin$ was formed.

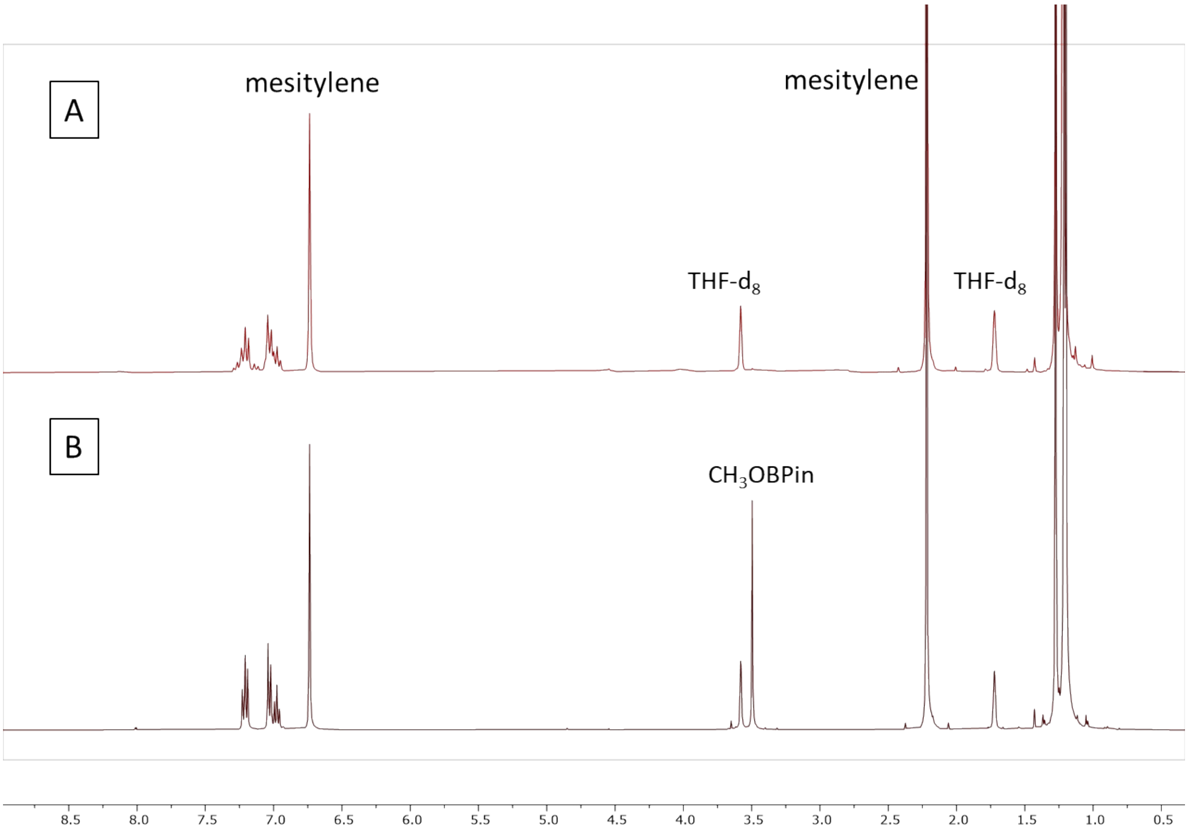


**Figure S5.** $^1H$ NMR spectra (400 MHz, THF-$d_8$) for $CO_2$ hydroboration (1 mol% **Co1** and 10 mol% $B(OPh)_3$ **A**: rt, 5 min (no conversion of HBpin). **B**: 60 °C, 18 h (full conversion of HBpin, 74% yield of $CH_3OBpin$).

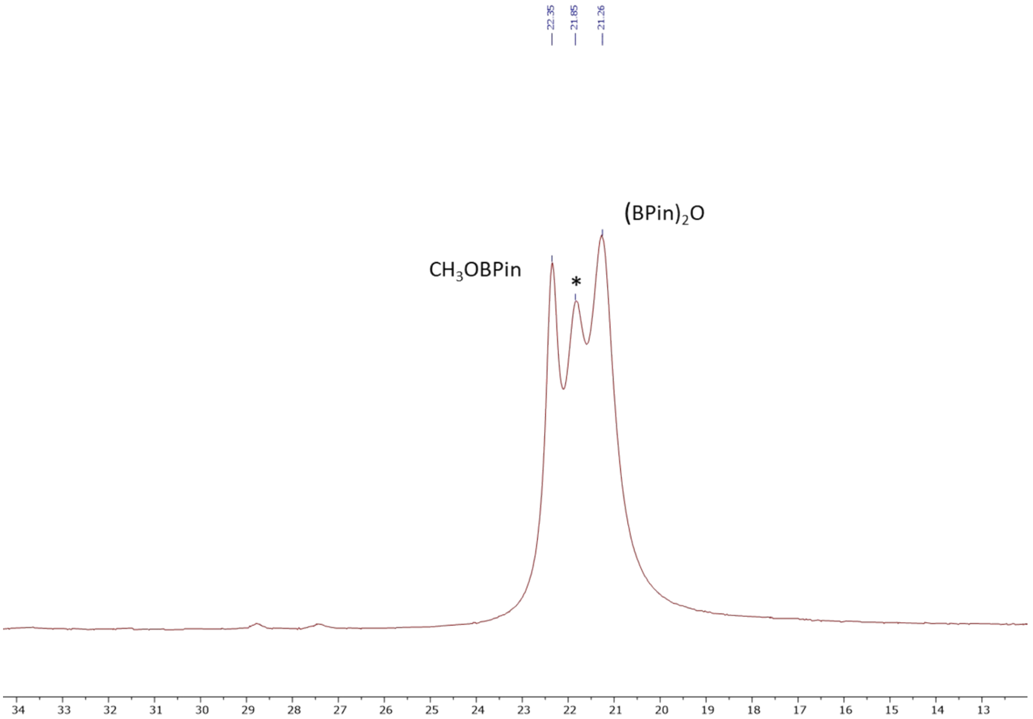


**Figure S6.** $^{11}$B NMR spectrum (400 MHz, THF-$d_8$) for $CO_2$ hydroboration (1 mol% **Co1** and 10 mol% $B(OPh)_3$) at 60 °C for 18 h; asterisks denote signals that belong to $B(OPh)_3$ or related species.

### *Shortened reaction time of the hydroboration of $CO_2$*

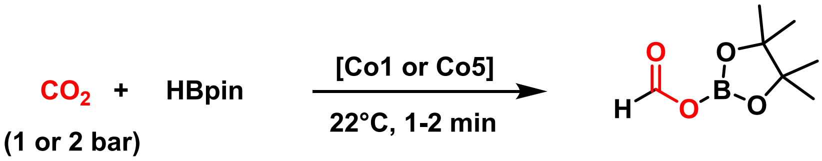


Four reactions were performed with complexes **Co1** and **Co5**, respectively, each at 1 and 2 min reaction times. Method A was slightly varied to allow facile purging with $CO_2$ and higher $CO_2$ pressure. Catalyst **Co1** or **Co5** (~0.85 mol%) were weighed in an inert vial equipped with a magnetic stir bar (5 mm) and THF-$d_8$ (0.6 mL) and mesitylene (15 µl, 0.108 mmol, internal standard) were added. The dark orange reaction solution was treated with HBpin (41 µl, 0.27 mmol) resulting in an immediate colour change to dark green. The vial was placed in the Parr Instr. stainless steel autoclave and pressurized with ~2 bar $CO_2$ for 1 or 2 min under stirring with an external magnetic stirring plate. Then, $CO_2$ was released, the autoclave opened and the vial retrieved. The reaction solution was transferred into an NMR tube under air. A colour change from yellow to purple was observed after contact to air. The yield of formyloxyborane was determined vs. the internal standard by $^1$H NMR (after 1 min: 90%; after 2 min: 99%).

NMR data of $HCO_2Bpin$:[7]

**$^1$H NMR** (400 MHz, THF-$d_8$) δ [ppm] = 8.40 (s, 1H, <u>H</u>$CO_2$Bpin), 1.31 (s, 12H, $HCO_2$B<u>pin</u>). **$^{11}$B NMR** (128 MHz, THF-$d_8$) δ [ppm] = 22.68.

**Calculated amount** of $CO_2$ $$n = \frac{pV}{RT}$$

Stainless steel autoclave by Parr Instr.: 2 bar n($CO_2$) ≈ 11.4 mol (vol. $CO_2$ ≈ 130 mL)

Activity and productivity of a 1 mol% [**Co1**] reaction at 20°C after 1 min with 90% conversion to product (by $^1$H NMR):

**TON** $\frac{n(product)\ 0.24\ mmol}{n(catalyst) 0.0023\ mmol} \approx 105$

**TOF** $\frac{TON}{t = 0.0166667h} \approx 6300\ h^{-1}$

The TON and TOF are minimal values due to the fast rate of the reaction and the determination from datapoints of full conversion.

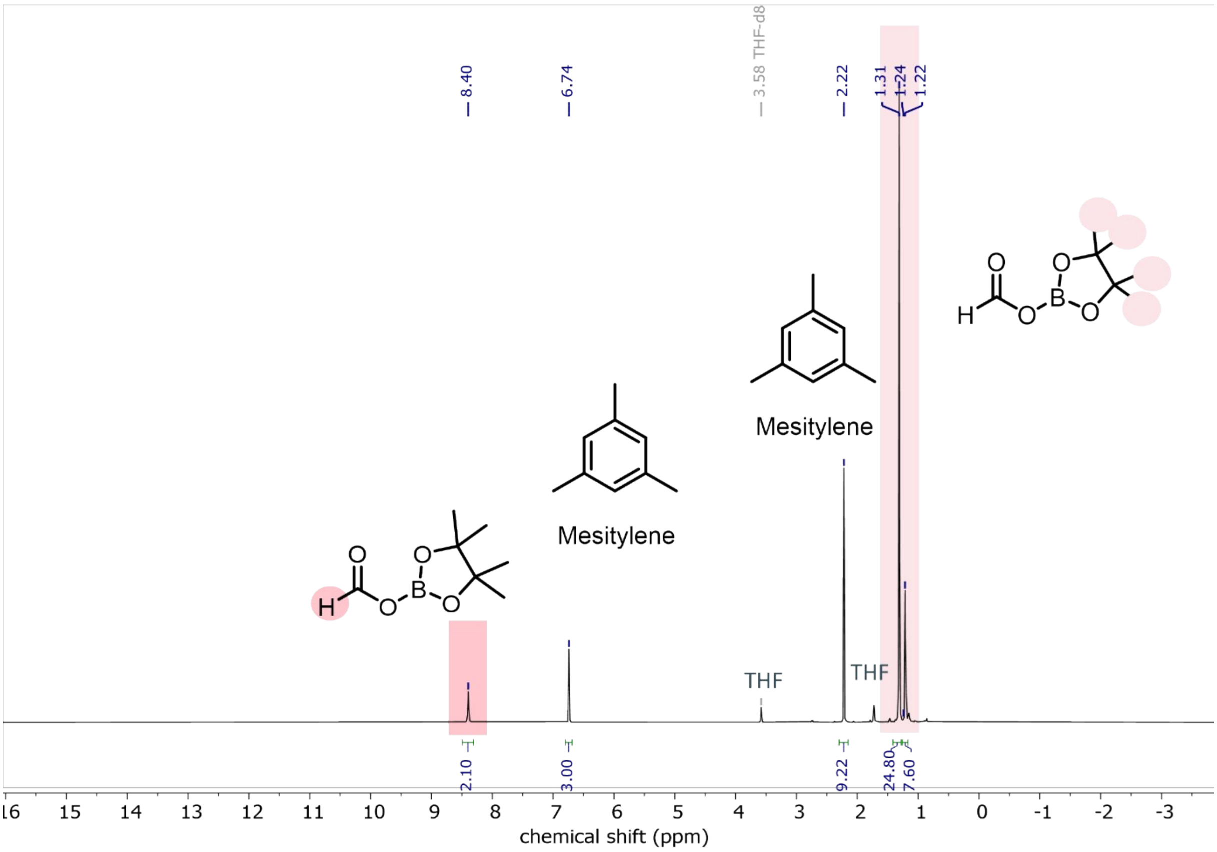


**Figure S7A.** $^1$H-NMR spectra (400 MHz, THF-$d_8$) for $CO_2$ hydroboration (0.85 mol% **Co1** and 2 bar $CO_2$, r.t., 1 min resulting in formyloxyborane (0.24 mmol, 90% yield).

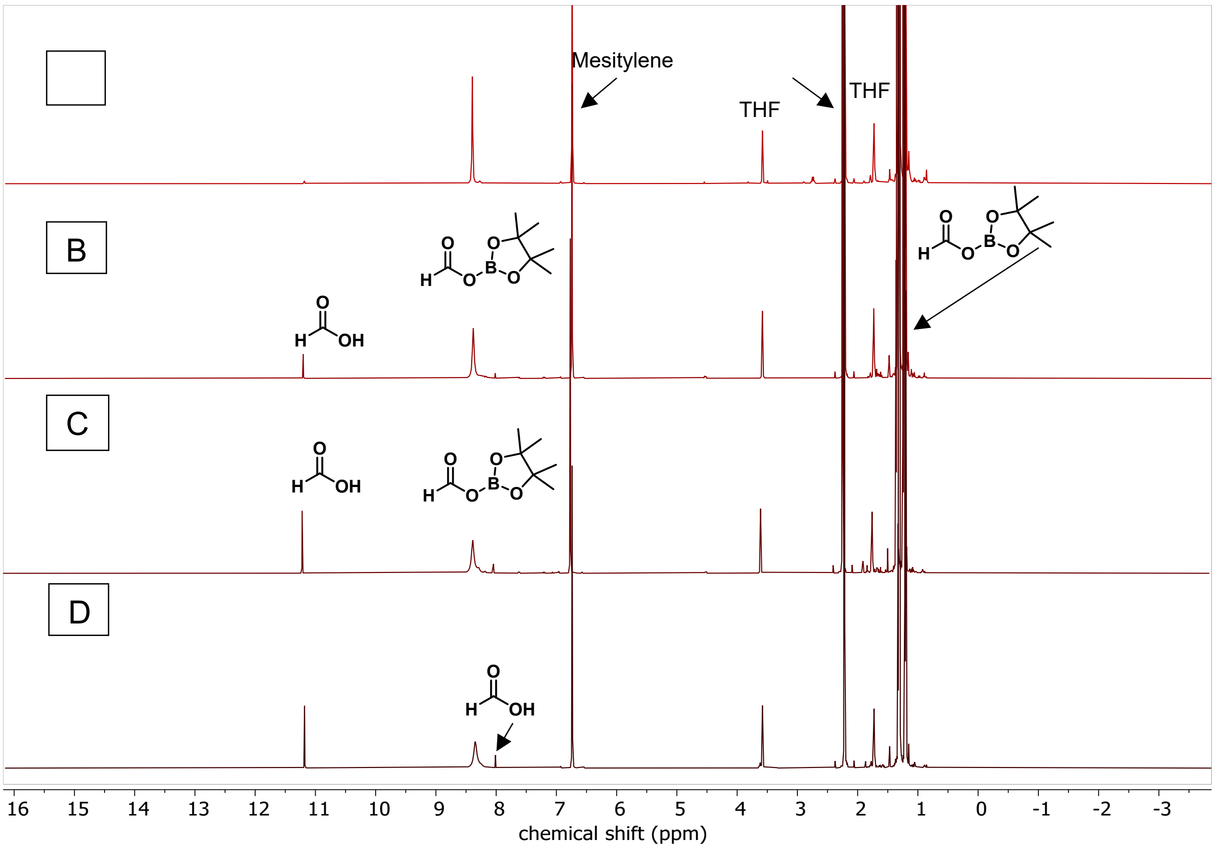


**Figure S7B.** $^1$H NMR spectra (300 MHz, THF-$d_8$): **A**) reaction solution with 0.85 mol% **Co1** was pressurized with 2 bar $CO_2$ for 1 min to full conversion $^1$H-NMR measured immediately under argon. **B)** reaction solution with 0.85 mol% **Co5** was pressurized with 2 bar $CO_2$ for 1 min. **C)** reaction solution with 0.85 mol% **Co1** was pressurized with 2 bar $CO_2$ for 2 min to full conversion $^1$H-NMR measured after 6 h, **D)** reaction solution with 0.85 mol% **Co1** was pressurized with 2 bar $CO_2$ for 2 min to full conversion $^1$H-NMR measured after 24 h. Spectra B-D were recorded after work-up under air, which resulted in partial hydrolysis of $HCO_2$Bpin to formic acid by moisture.

### *Preparative-scale N-formylation of aniline in presence of **Co1***

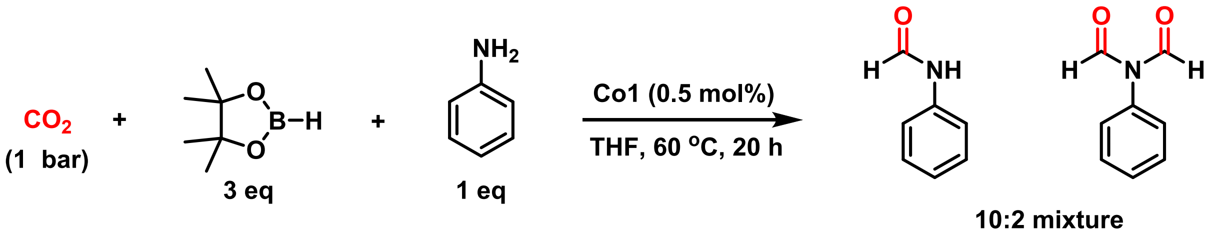


The 10 mL Teflon-cock Schlenk tube was charged with **Co1** (5.2 mg, 0.0125 mmol), aniline (76 µL, 0.833 mmol) and 3 mL THF. Pinacolborane (373 µL, 2.5 mmol, 3 equiv.) was added to reaction mixture at room temperature. Intensive gas evolution and colour change from orange to brown was observed. Then, the Schlenk tube was sealed, and

solution was degassed with two freeze-pump-thaw cycles. Then, the solution was immersed into a liquid nitrogen bath for the third time and vacuum was applied to the frozen solution. The Schlenk tube was removed form the liquid nitrogen, reaction mixture was exposed to 1 atm of $CO_2$ for 5 min. Then, the Schlenk-tube was sealed and reaction mixture was stirred for 16 h at 60 °C to give a colorless solution. All volatiles were removed under vacuum and the residue was purified by column chromatography on silica gel using $CH_2Cl_2$ as eluent. Yield: 87 mg (86%). The isolated product constitues the unseparated mixture of N-formamide / N-formyl-N-phenylformamide = 10/2. NMR data is in agreement with previous literature reports.[9,10]

**N-formanilide** is a mixture of rotamers (major and minor).

**$^1$H NMR** (600 MHz, $CDCl_3$): δ [ppm] = 8.70 (d, *J* = 11.3 Hz, 1H, major rotamer), 8.57 (br.s, 0.9H, major rotamer), 8.36 (d, *J* = 1.9 Hz, 0.8H, minor rotamer), 7.65 (br. s, 0.8H, minor rotamer), 7.57 – 7.53 (m, 1.7H, minor + major rotamer), 7.39 – 7.30 (m, 3.7H, minor + major rotamer), 7.20 – 7.17 (m, 1H, major rotamer), 7.15 – 7.12 (m, 0.8H, minor rotamer), 7.11 – 7.08 (m, 1.8H, minor + major rotamer). **$^{13}$C NMR** (75 MHz, $CDCl_3$, 25 °C) δ [ppm] = Major rotamer: 162.72, 136.81, 129.90, 125.44, 118.97 Minor rotamer: 159.11, 136.97, 129.25, 124.97, 120.10.

*N-formyl-N-phenylformamide*

**$^1$H NMR** (600 MHz, $CDCl_3$, 25 °C) δ [ppm] = 9.10 (br s), 7.50 (d, *J* = 16.2 Hz, 3H), 7.23 (br. d, *J* = 7.7 Hz, 2H).

### *NMR-scale N-formylation of aniline in presence of* ***Co1***

The J. Young NMR tube was charged with **Co1** (1.1 mg, 2.7 µmol), aniline (8 µL, 0.09 mmol) and 0.6 mL THF-$d_8$. Pinacolborane HBpin (41 µL, 0.27 mmol) was added to the reaction mixture and vigorous gas evolution was observed for few seconds after addition. The J. Young NMR tube was sealed and attached to a $CO_2$ gas supply line. The reaction mixure was degassed with two freeze-pump-thaw cycles. Then, the solution was immersed into a liquid nitrogen bath for the third time and vacuum was applied to the frozen solution. The J. Young NMR tube was lifted form the liquid nitrogen and reaction mixture was exposed to 1 bar $CO_2$ for 5 min. The J. Young tube was then sealed, shaken vigorously and placed to the 60 °C oil bath. Reaction progress was monitored by $^1$H NMR.

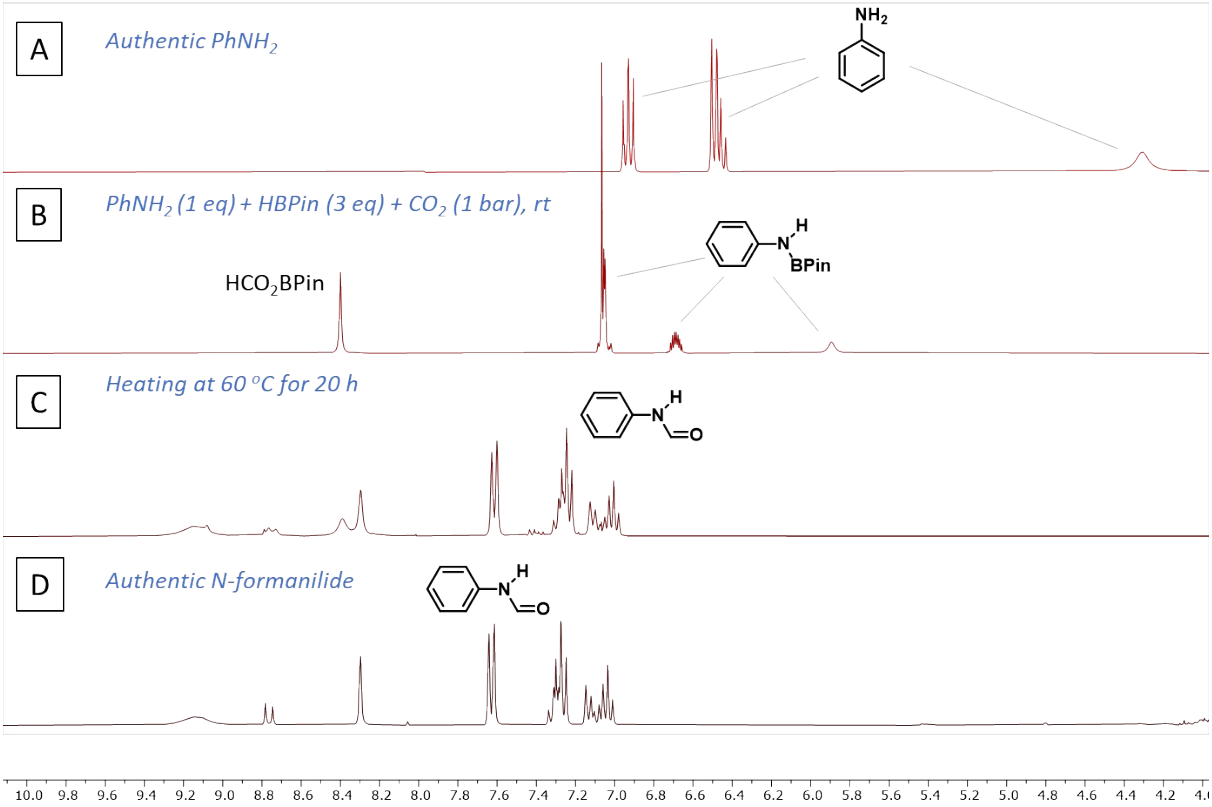


**Figure S8.** $^1$H NMR spectra (300 MHz, THF-$d_8$): **A**) Pure aniline in THF-$d_8$; **B**) Mixture of aniline (1 equiv.) and HBpin (3 equiv.) in presence of 1 mol% **Co1** after $CO_2$ addition for 5 min showing formation of 2:1 mixture of $HCO_2Bpin$ and dehydrocoupling product PhNHBpin[11]; **C**) heating at 60 °C for 20 h showing formation of N-formanilide as predominant product; **D**) pure N-formanilide in THF-$d_8$ for reference.

## $CO_2$ hydroboration with different boranes

*General procedure:* Catalyst **Co1** (1.1 mg, 2.7 µmol), internal standard mesitylene (15 µL, 0.1078 mmol) and 0.6 mL THF-$d_8$ were mixed in a scintillation vial. The borane of choice (0.27 mmol) was added and reaction mixure was transferred to a J. Young NMR tube. The reaction mixture was degassed with two freeze-pump-thaw cycles. Then, the solution was immersed into a liquid nitrogen bath for the third time and vacuum was applied to the frozen solution. The J. Young NMR tube was lifted form the liquid nitrogen and reaction mixture was exposed to 1 atm of $CO_2$ for 5 min. The J. Young NMR tube was then sealed, shaken vigorously and subjected to $^1H$ and $^{11}B$ NMR analysis. $^1H$ NMR yield was determined by integration of signals of $CO_2$ hydroboration products with reference to the internal standard mesitylene.

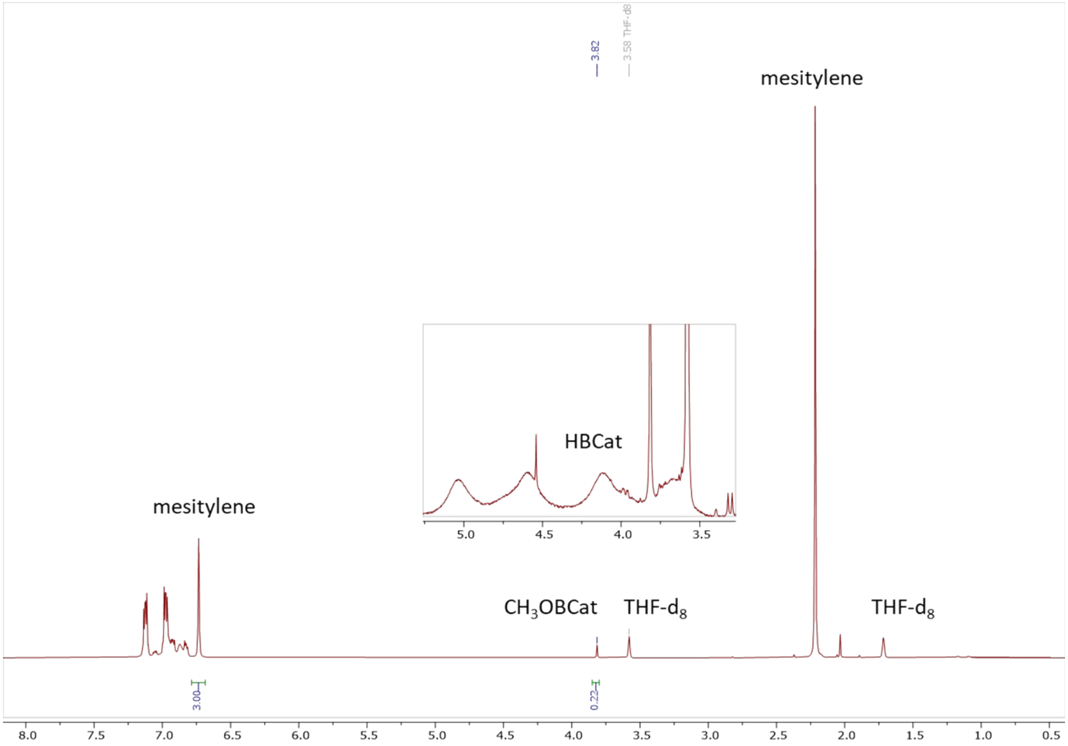


**Figure S9.** $^1H$ NMR spectrum (400 MHz, THF-$d_8$) of $CO_2$ hydroboration with HBcat. BH resonance of unreacted HBcat is shown in the inset. Spectral data is in accordance with the literature.[7]

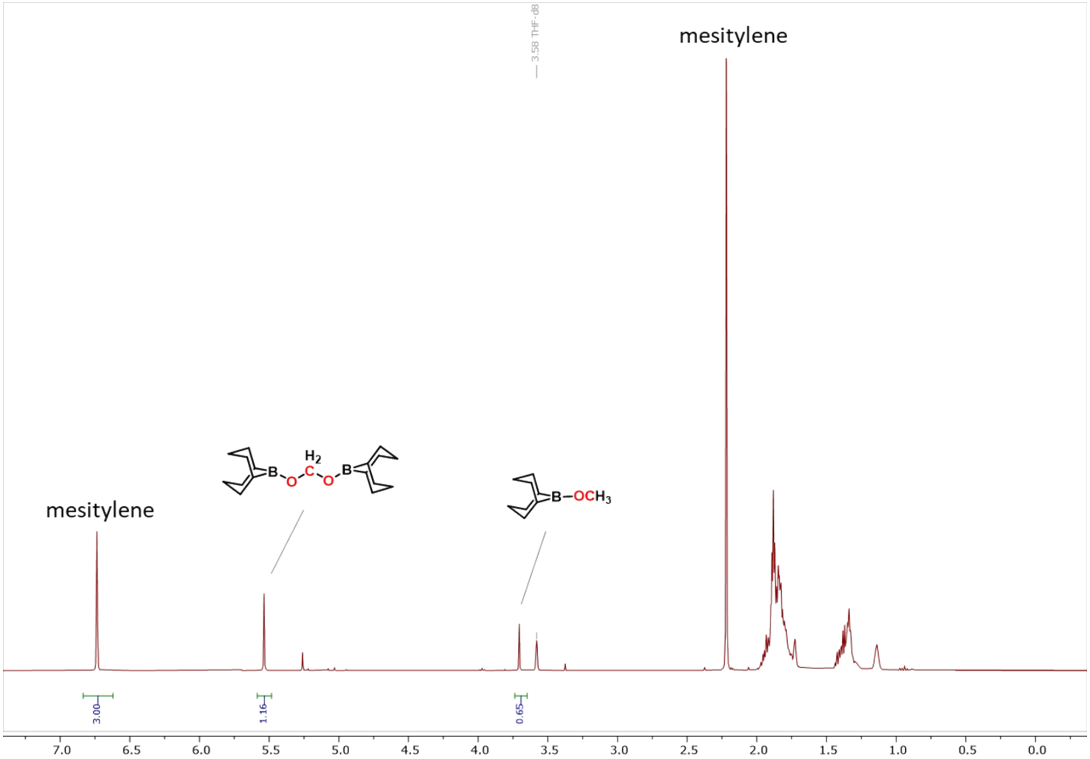


**Figure S10.** $^{1}H$ NMR spectrum of $CO_2$ hydroboration with HBcat. Spectral data is in accordance with the literature.[7]

## Mechanistic Experiments

*In situ generation of **L1Bpin***

The J. Young NMR tube was charged with **L1H** (15 mg, 67 µmol), HBpin (10 µL, 67 µmol) and 0.6 mL THF-$d_8$ and the reaction mixture was heated at 60 °C for 16 h. Clean conversion of starting materials to form the coupling product **L1Bpin** was proved by $^1$H, $^{11}$B and $^{31}$P spectra. Aliquots of the obtained solution were used to test background catalytic activity of **L1Bpin**.

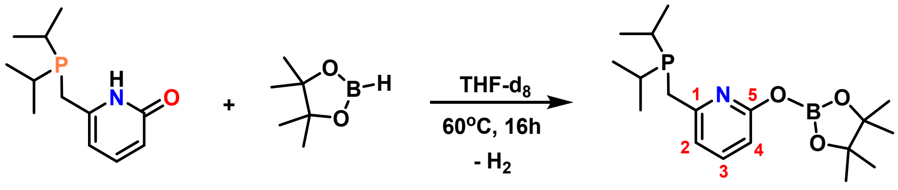


**L1H** **L1BPin**

**$^1$H NMR** (400 MHz, THF-$d_8$) δ [ppm] = 7.52 (t, *J* = 7.7 Hz, 1H, H-3), 6.97 (d, *J* = 7.5 Hz, 1H, H-2), 6.62 (d, *J* = 8.1 Hz, 1H, H-4), 2.86 (br.s, 2H, C$\underline{H}_2$-P), 1.80 (m, 1H, *i*-Pr-C$\underline{H}$), 1.28 (s, 12H, Bpin-C$\underline{H}_3$), 1.12 – 0.99 (m, 12H, *i*-Pr-C$\underline{H}_3$) **$^{13}$C NMR** (75 MHz, THF-$d_8$) δ [ppm] = 159.63 (C5), 159.39 (C1), 138.82 (C3) 118.20 (C2), 109.35 (C4), 82.96 (C-BPin), 31.55 (d, $^1J_{C,P}$ = 23.9 Hz, $\underline{C}H_2$Py), 23.91 (Bpin-$\underline{C}H_3$ overlap with THF-$d_8$), 23.36 (d, $^3J_{C,P}$ = 16.1 Hz), 19.17 (d, *J* = 15.5 Hz, *i*-Pr-*C*$H_3$), 18.53 (d, $^3J_{C,P}$ J = 11.4 Hz, *i*-Pr-*C*$H_3$) **$^{31}$P NMR** (162 MHz, THF-$d_8$) δ [ppm] = 12.07 **$^{11}$B NMR** (128 MHz, THF-$d_8$) δ [ppm] = 22.10.

*Stoichiometric reaction between **Co1** and Hbpin* [6]

HBpin (6 µL, 0.40 mmol) was added to the solution of **Co1** (16 mg, 0.38 mmol) in 0.6 mL THF-$d_8$ resulting in instant color change from orange-red to black. The reaction mixture was stirred overnight at ambient temperature and transferred to J. Young NMR tube. $^1$H and $^{11}$B NMR spectra were recorded. $^{11}$B NMR spectrum showed complete consumption of HBpin. $^1$H NMR spectrum showed a mixture of paramagnetic and diamagnetic compounds.

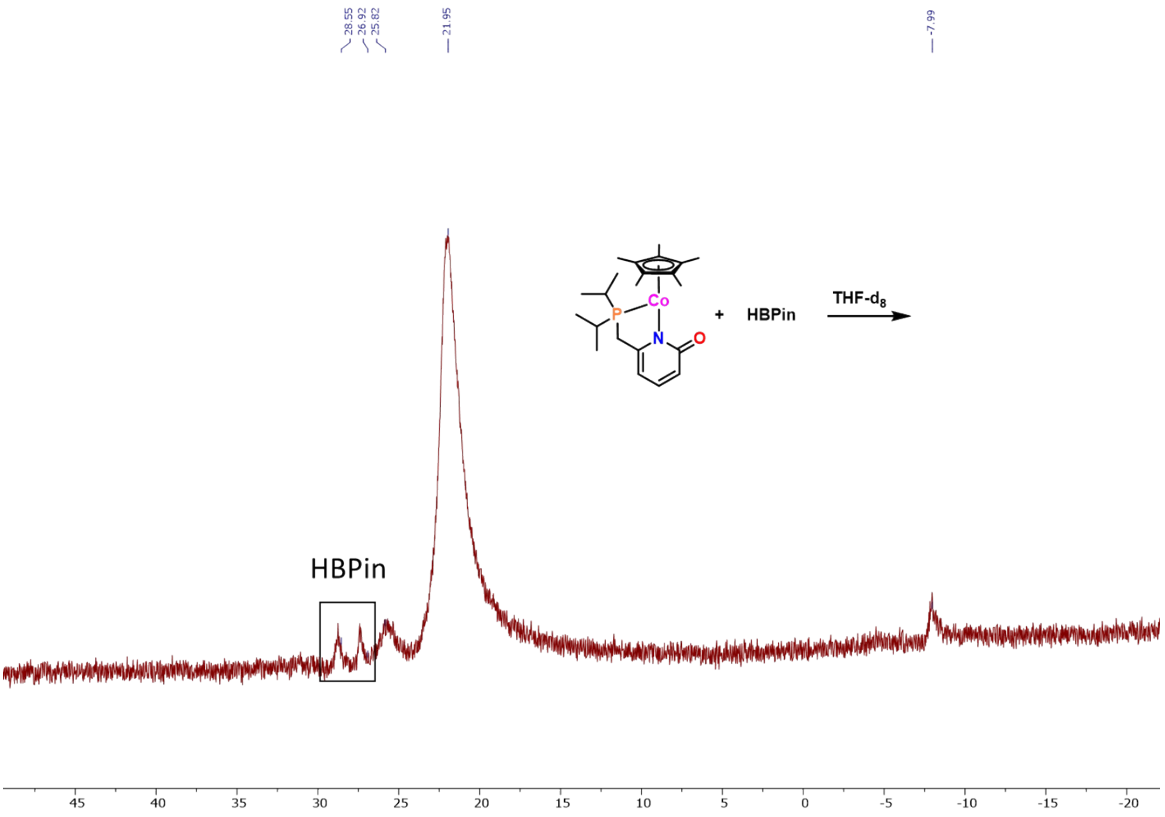


**Figure S11.** $^{11}$B NMR spectrum (400 MHz, THF-$d_8$) of **Co1** + HBpin reaction mixture showing almost full consumption of HBpin and formation of new species.

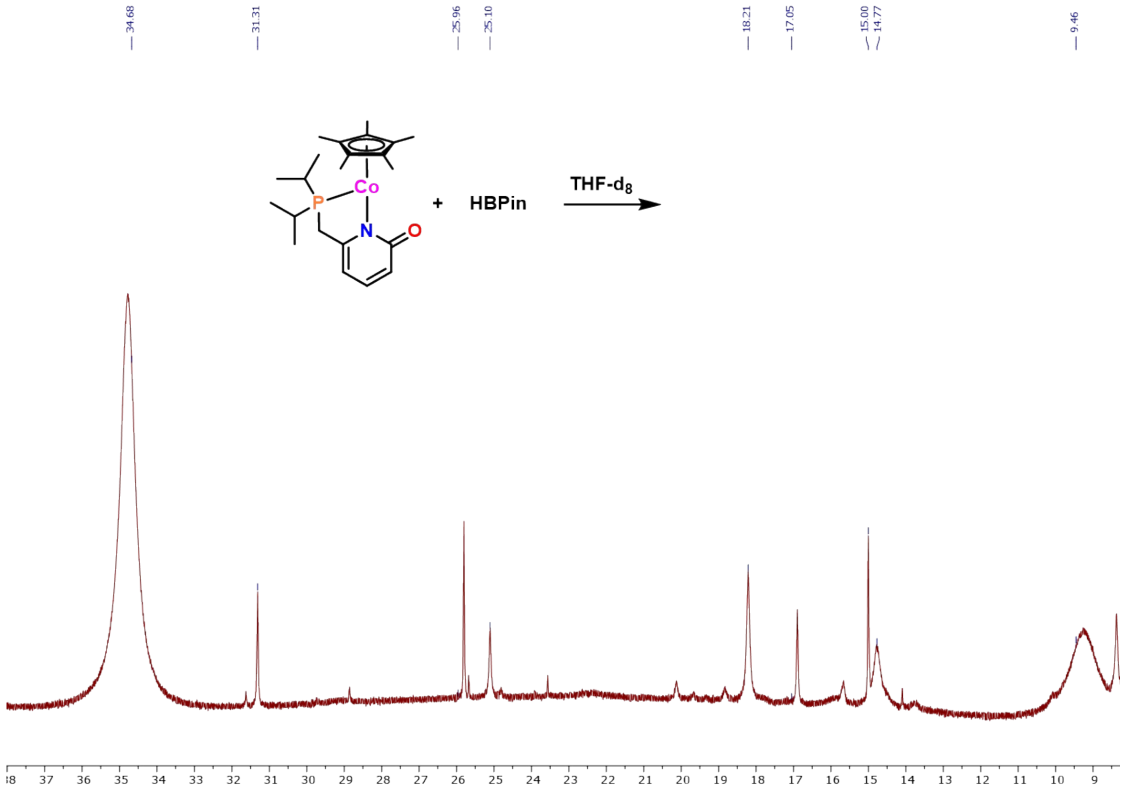


**Figure S12.** Fragment of [1]H NMR spectrum (600 MHz, THF-$d_8$) of **Co1** + HBpin reaction mixture showing paramagnetically shifted resonances (not assigned).

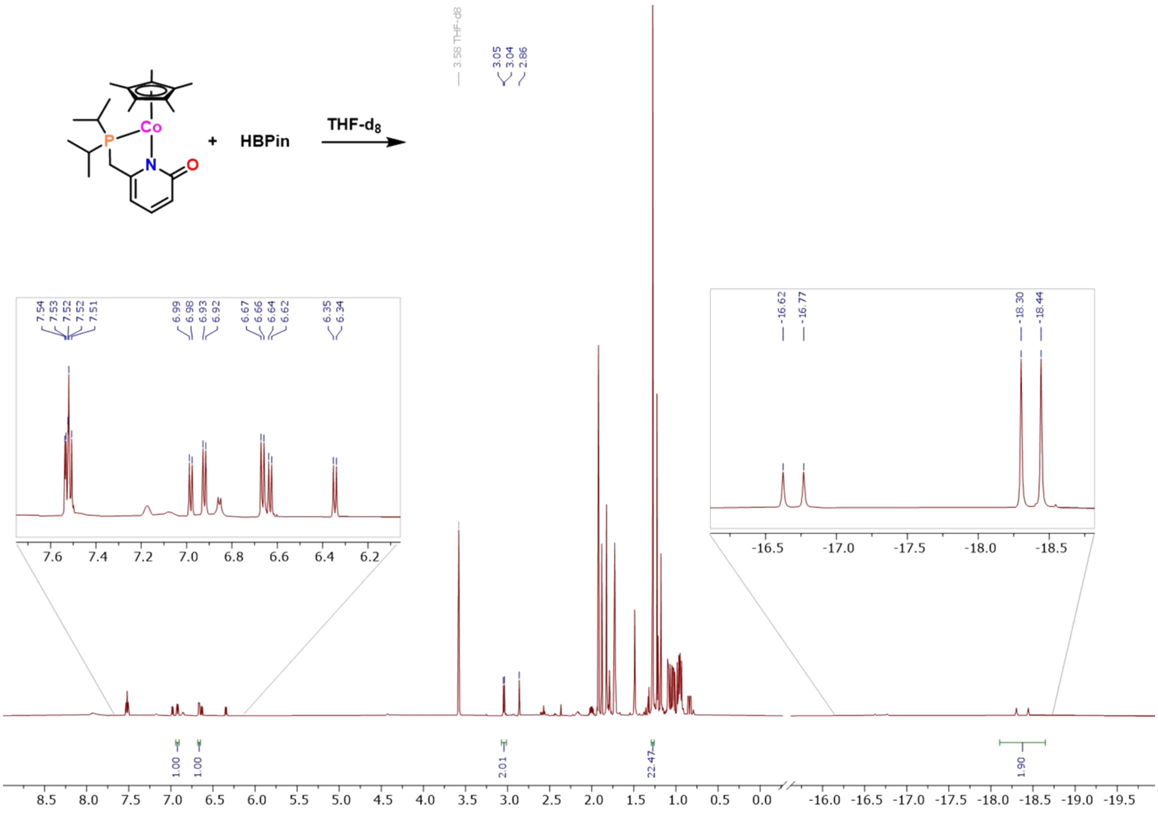


**Figure S13.** Diamagnetic region of the [1]H spectrum (600 MHz, THF-$d_8$) of the **Co1** + HBpin mixture.

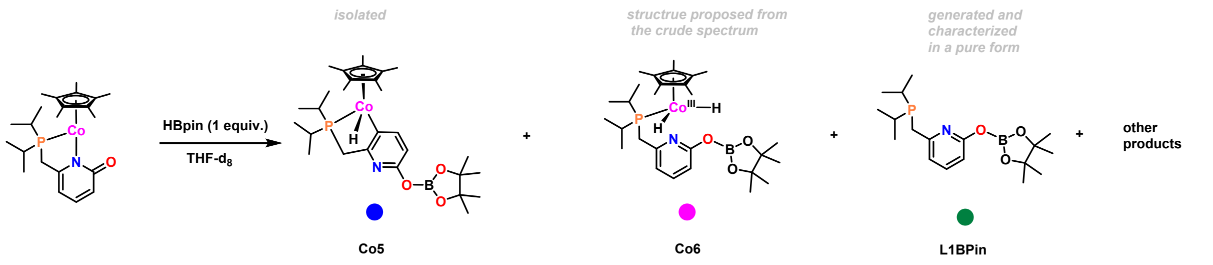


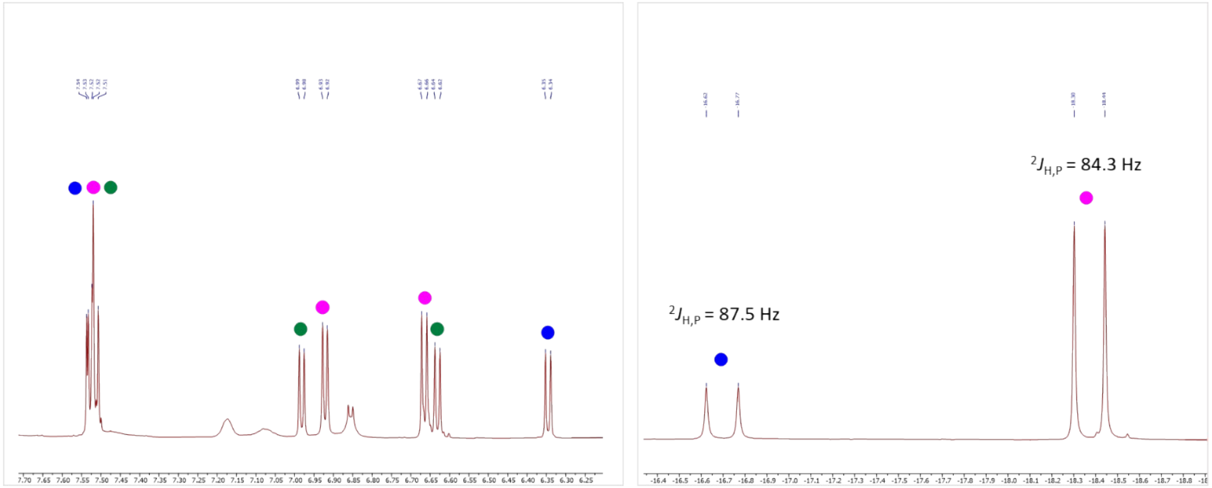


**Figure S14.** $^1$H NMR spectrum (600 MHz, THF-d$_8$) of the **Co1** + HBpin mixture with assignments.

*Stoichiometric reaction between **Co1** and DBpin* [6]

The aliquote of the 1.22 M stock solution of DBpin (47 μL, 57 μmol) was added to the solution of **Co1** (20 mg, 48 μmol). The reaction mixture was transferred to J. Young NMR tube and analyzed by $^1$H NMR spectra.

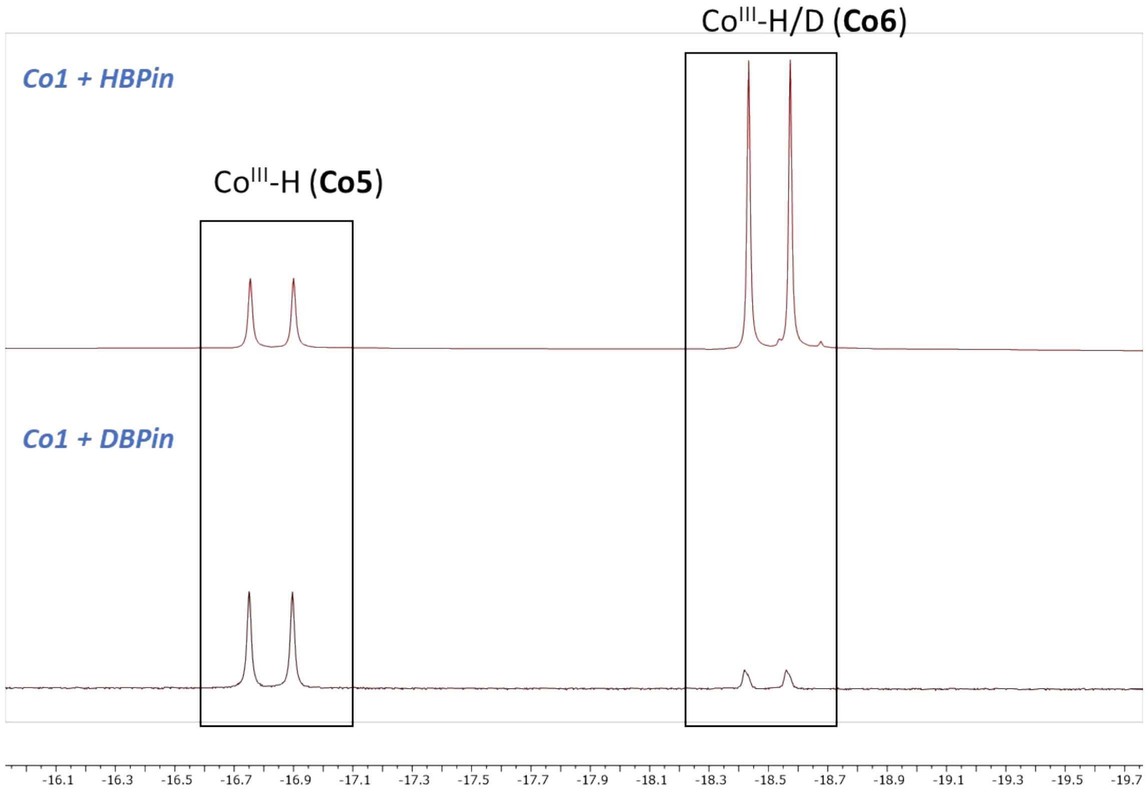


**Figure S15.** The upfield region of $^1$H NMR spectrum of the reaction mixture of **Co1** and HBpin (top); **Co1** and DBpin (bottom).

## NMR Spectra

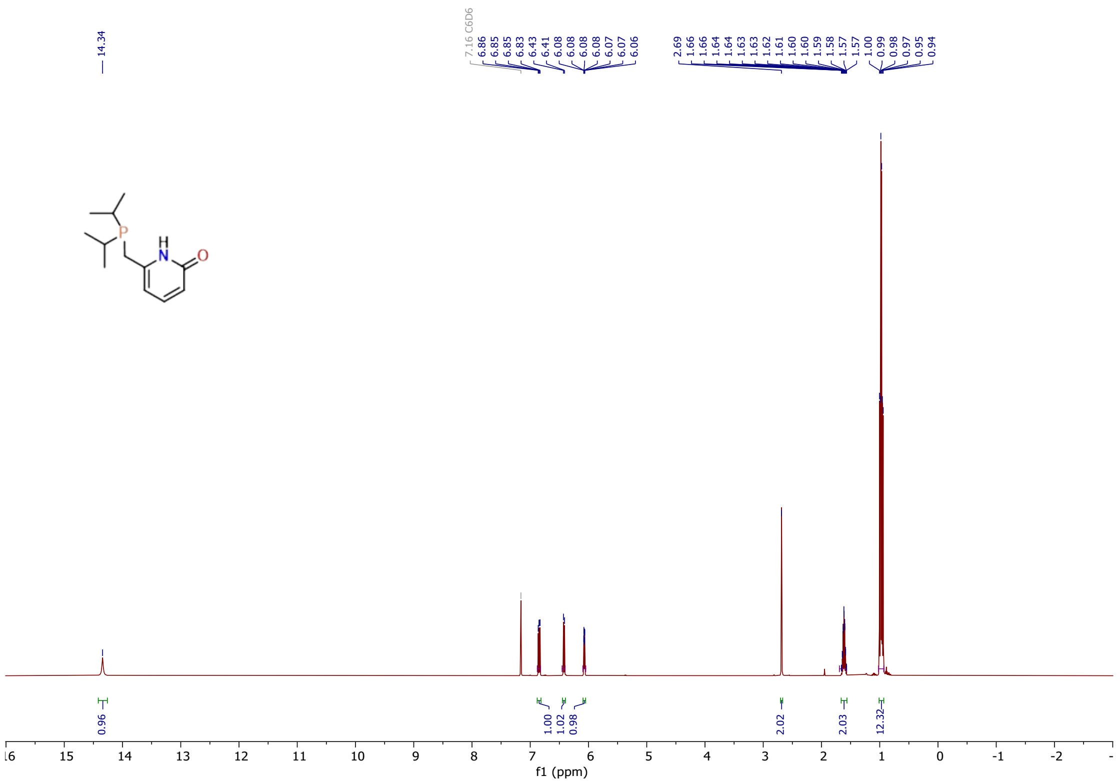

**Figure S16.** $^{1}H$ NMR spectrum (500.1 MHz, $C_6D_6$) of **L1H**.

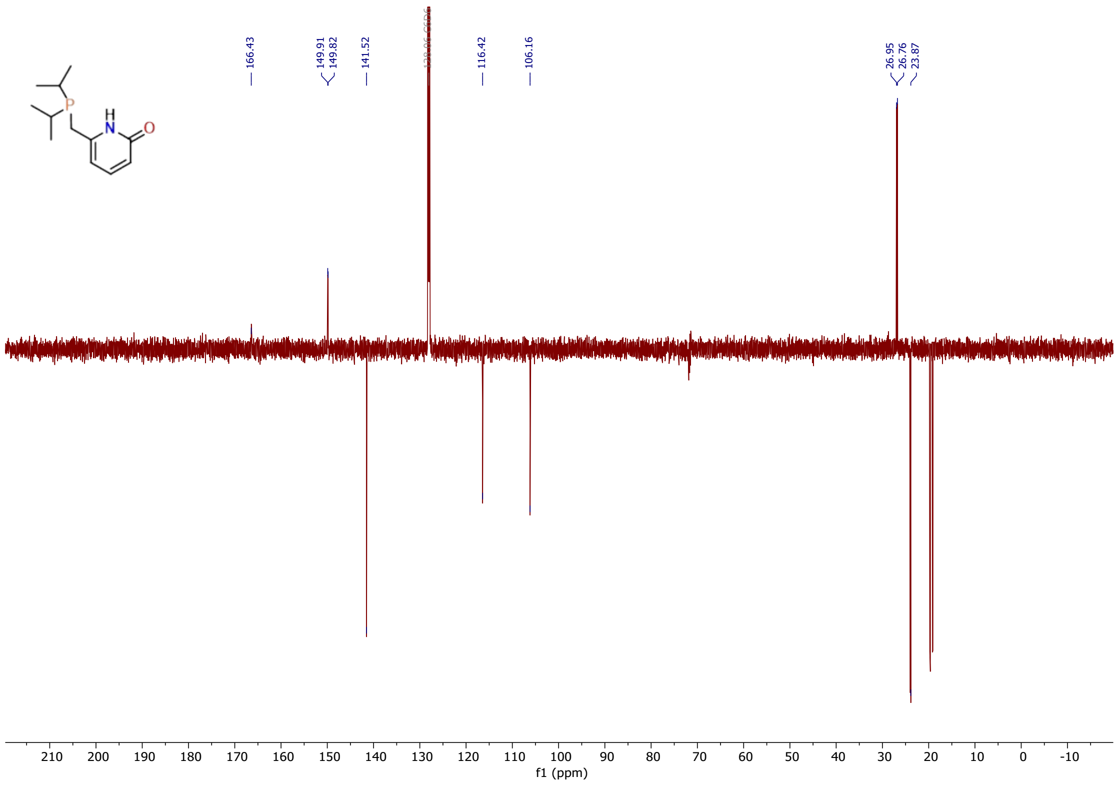

**Figure S17.** $^{13}C$-DEPTQ NMR spectrum of (125.8 MHz, $C_6D_6$) of **L1H**.

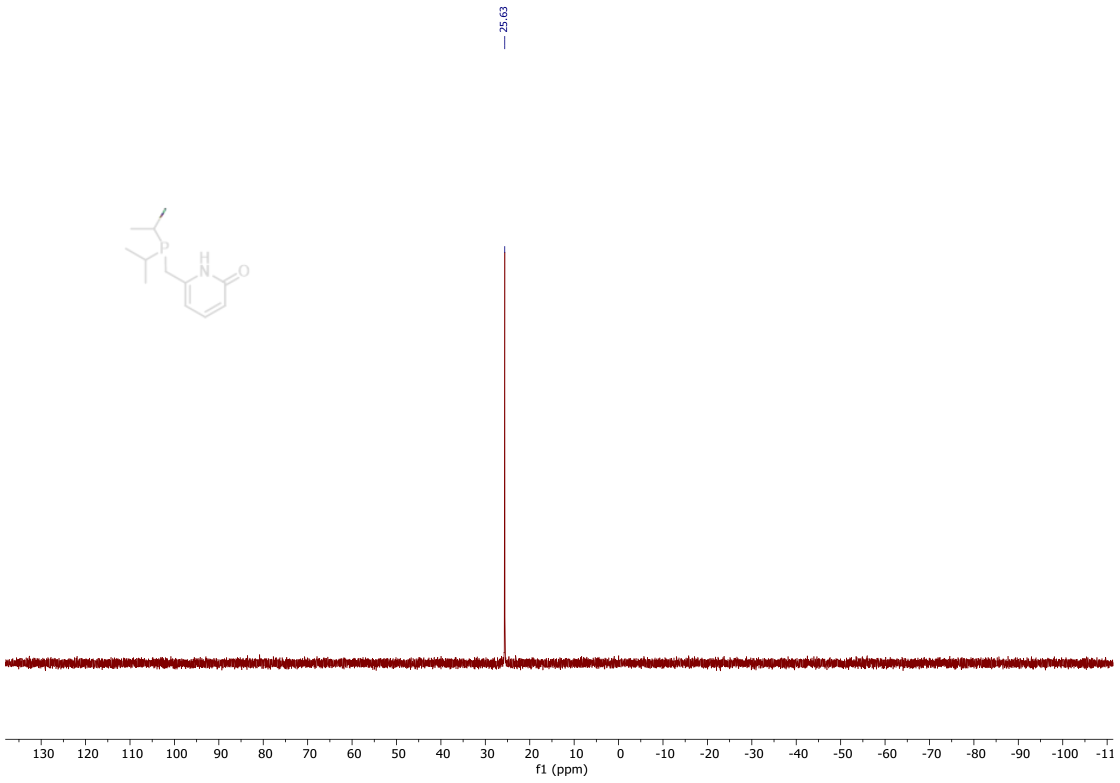


**Figure S18.** $^{31}P\{^{1}H\}$-NMR (202.5 MHz, $C_6D_6$) of **L1H**.

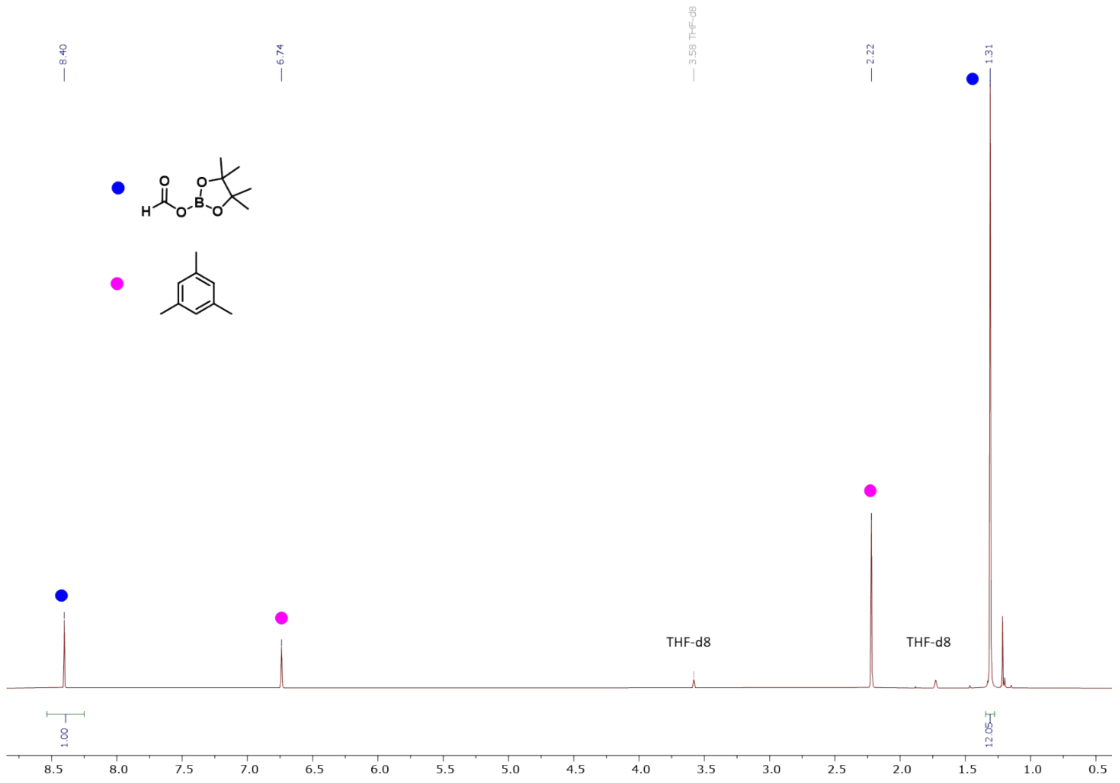


**Figure S19.** $^{1}H$ NMR spectrum (400 MHz, THF-$d_8$) of the crude product mixture of $CO_2$ hydroboration.

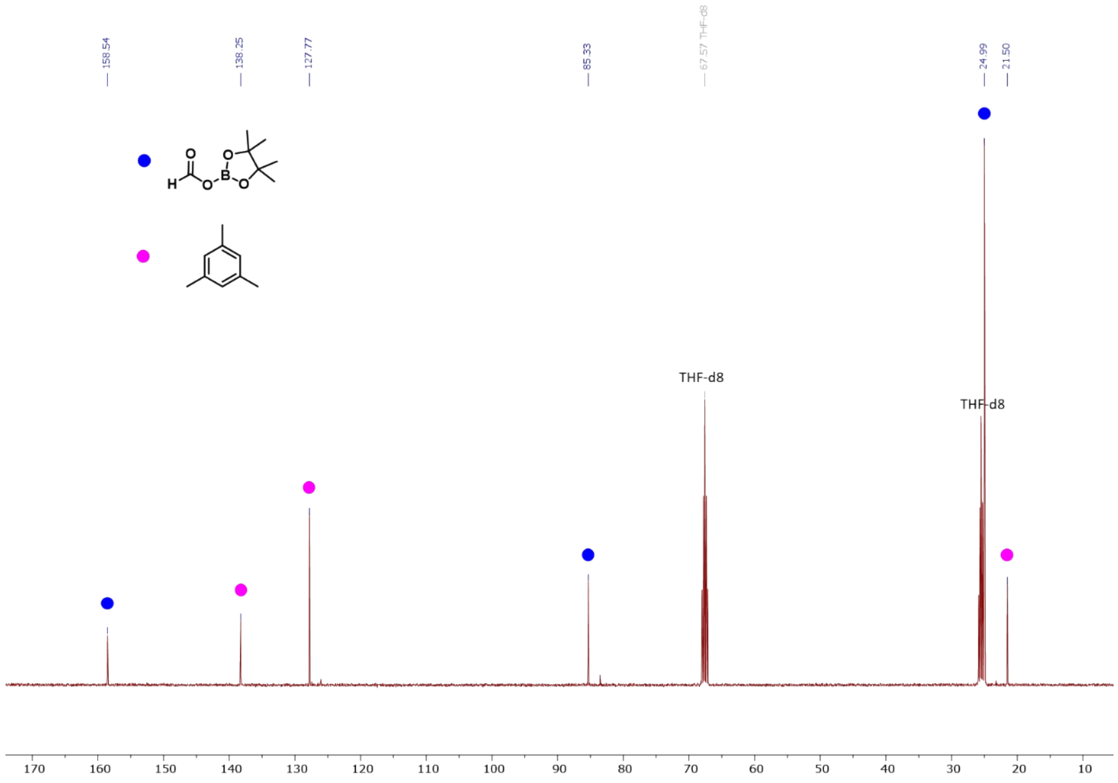


**Figure S20.** $^{13}C$ NMR spectrum (400 MHz, THF-$d_8$) of the crude product mixture of $CO_2$ hydroboration.

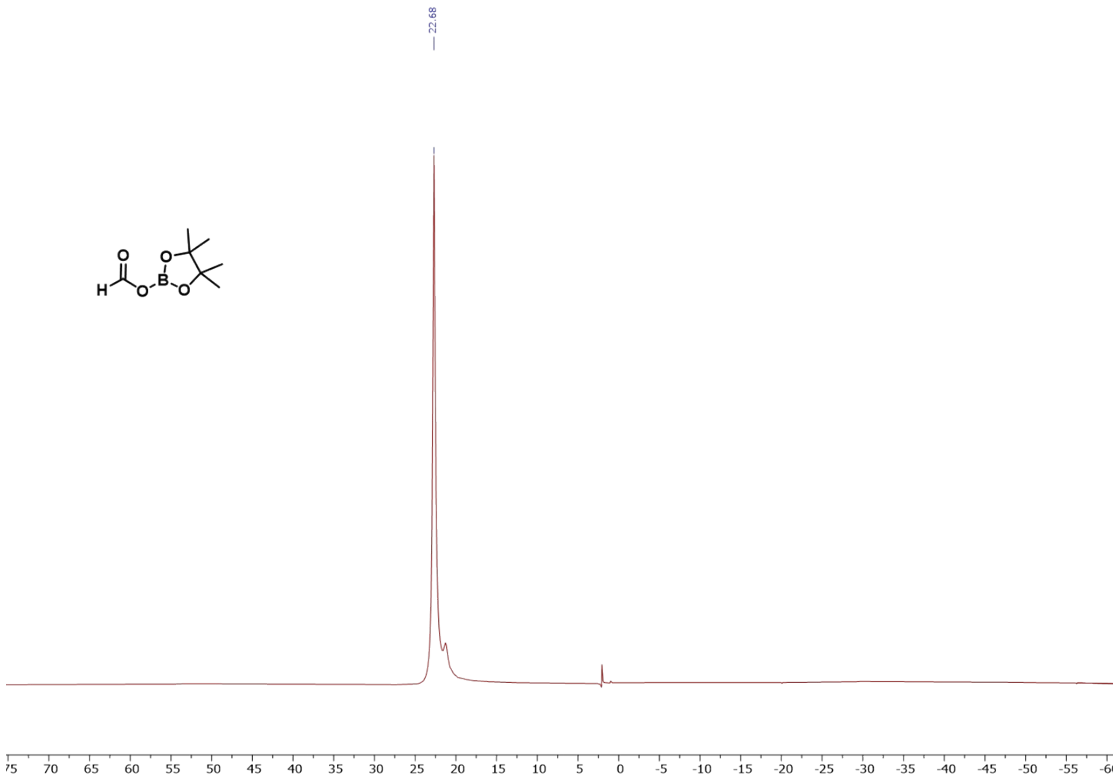


**Figure S21.** $^{11}B$ NMR spectrum (400 MHz, THF-$d_8$) of the crude product mixture of $CO_2$ hydroboration.

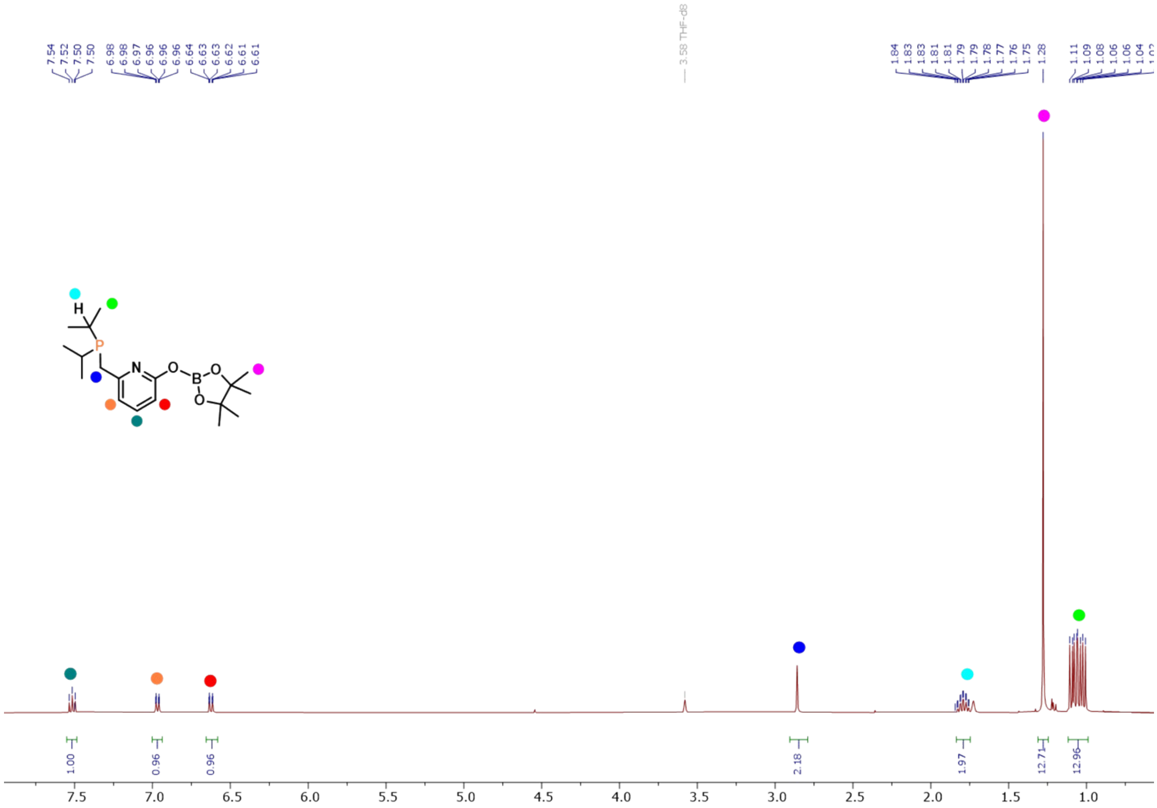

**Figure S22.** $^{1}$H NMR spectrum (400 MHz, THF-$d_8$) of *in situ* generated **L1Bpin**.

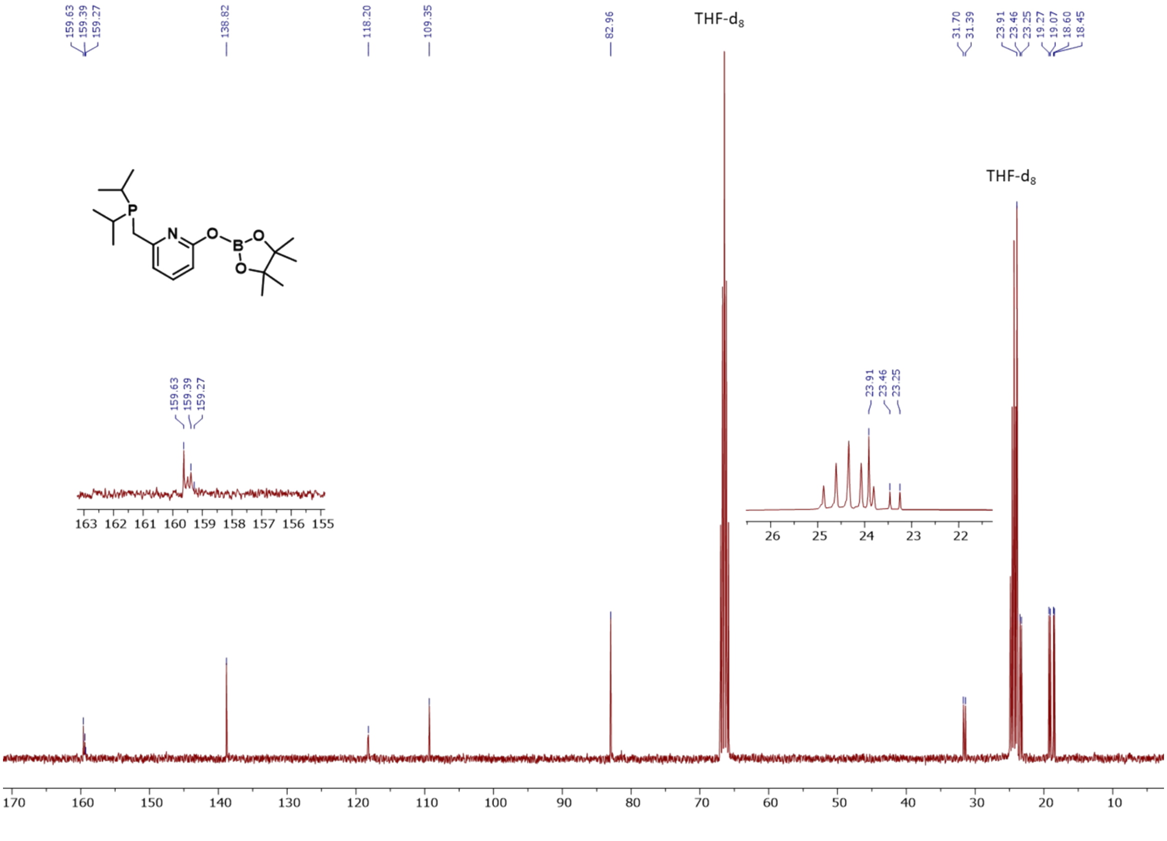

**Figure S23.** $^{13}$C NMR spectrum (300 MHz, THF-$d_8$) of *in situ* generated **L1Bpin**.

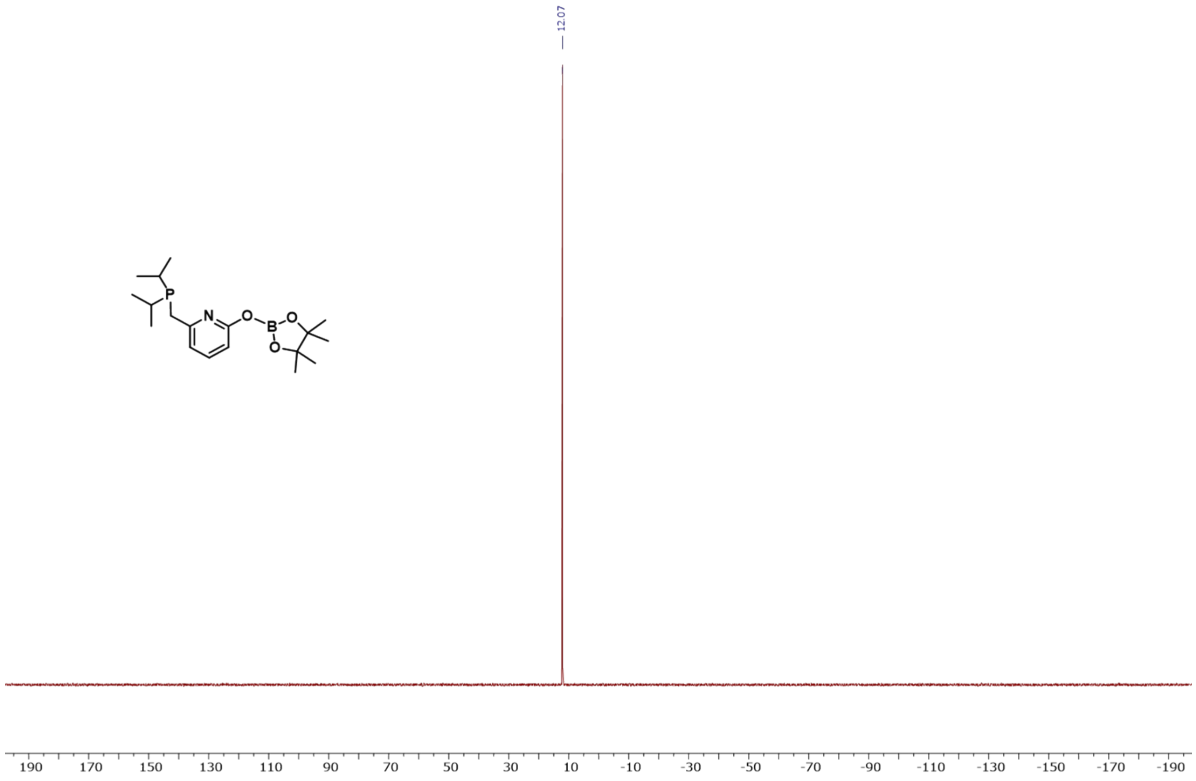


**Figure S24.** $^{31}P$ NMR spectrum (162 MHz, THF-$d_8$) of *in situ* generated **L1Bpin**.

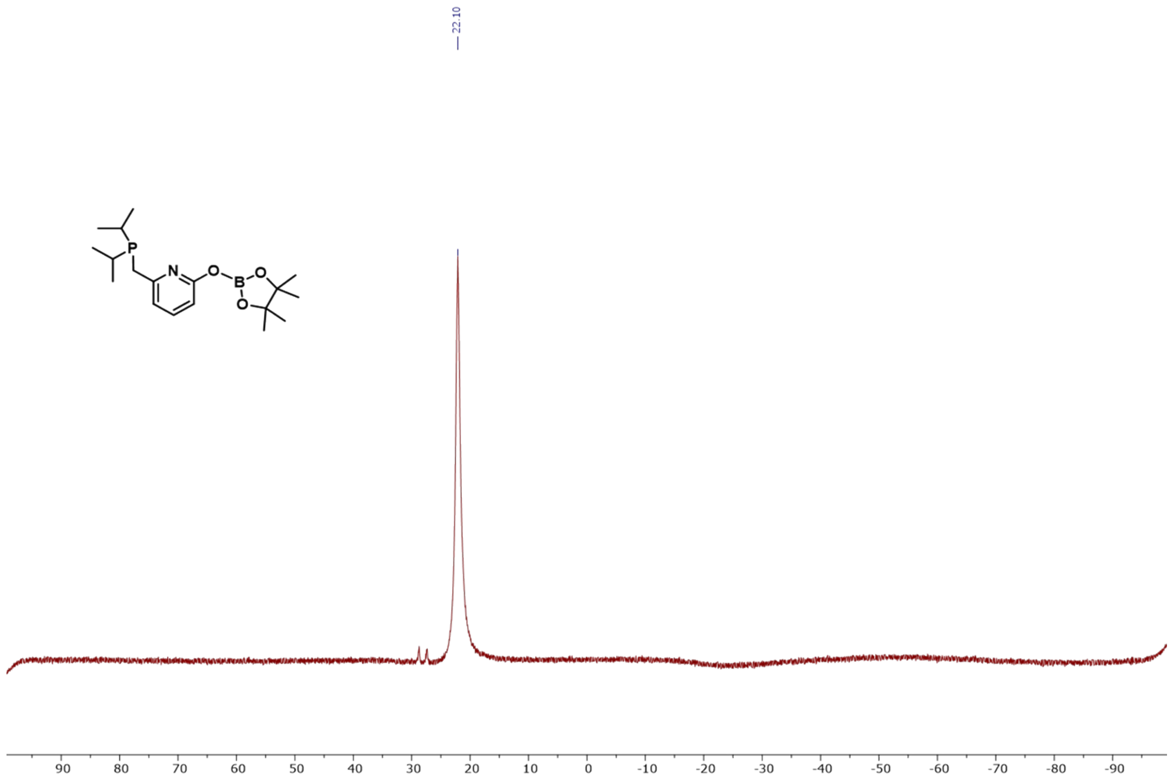


**Figure S25.** $^{1}H$ NMR spectrum (128 MHz, THF-$d_8$) of *in situ* generated **L1Bpin**.

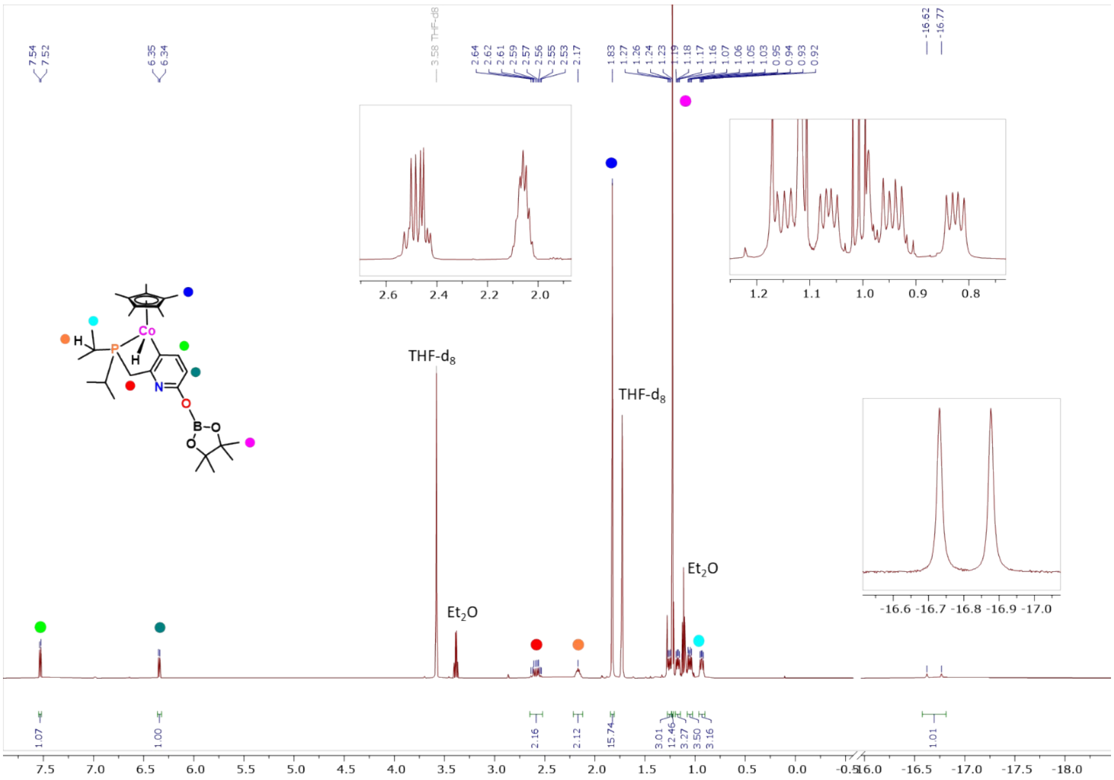

**Figure S26.** $^{1}H$ NMR spectrum (600 MHz, THF-$d_8$) of **Co5**.

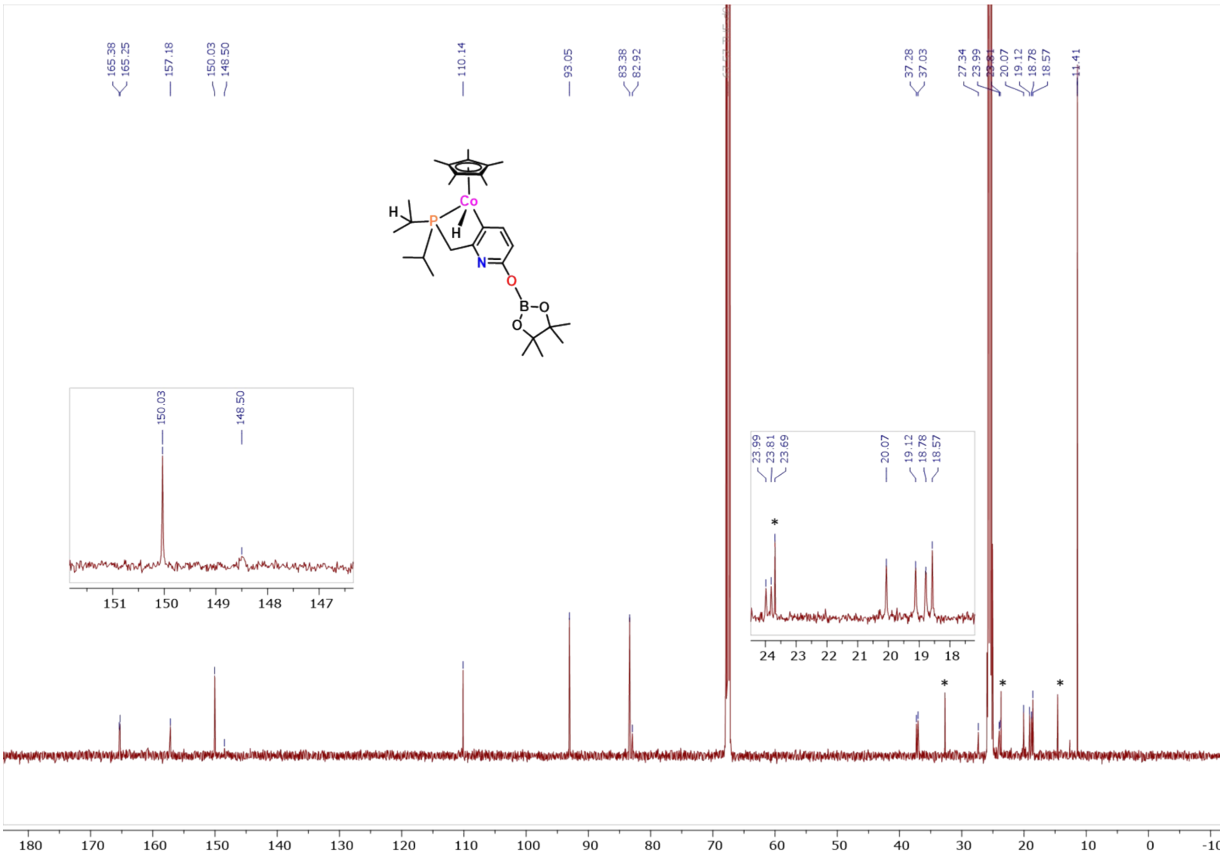

**Figure S27.** $^{13}C$ NMR spectrum (151 MHz, THF-$d_8$) of **Co5**. Asterisk denotes residual hexane.

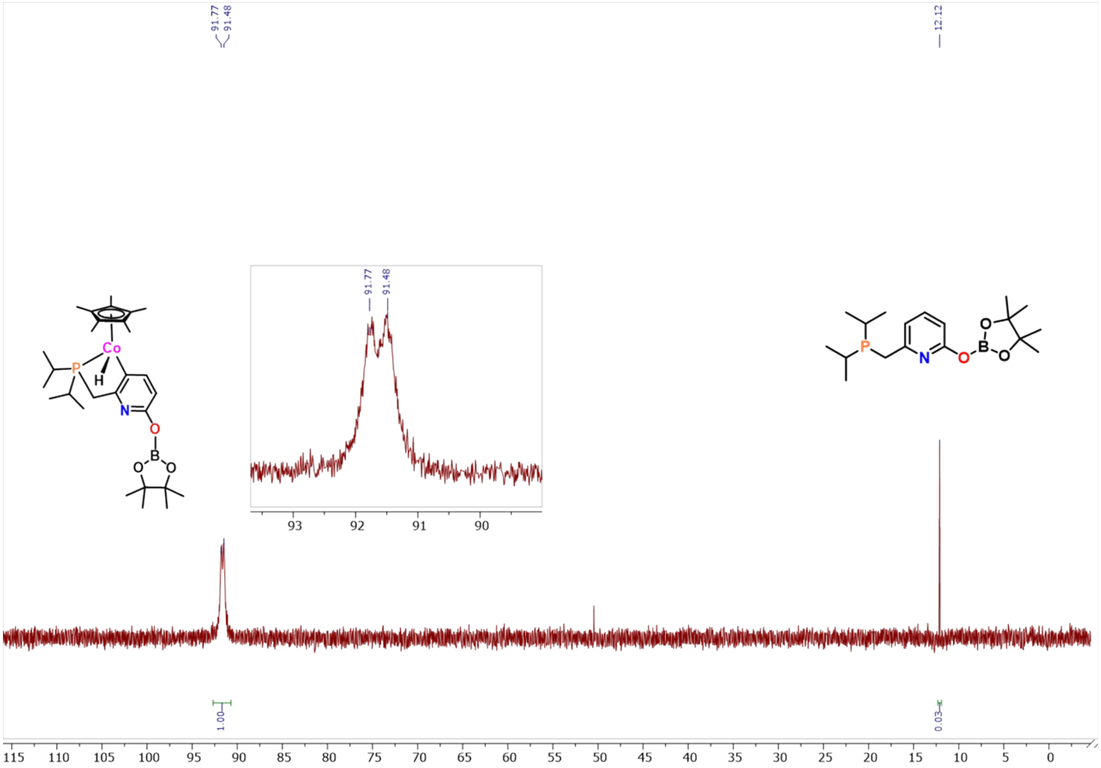

**Figure S28.** $^{31}P$ NMR spectrum (243 MHz, THF-$d_8$) of **Co5**.

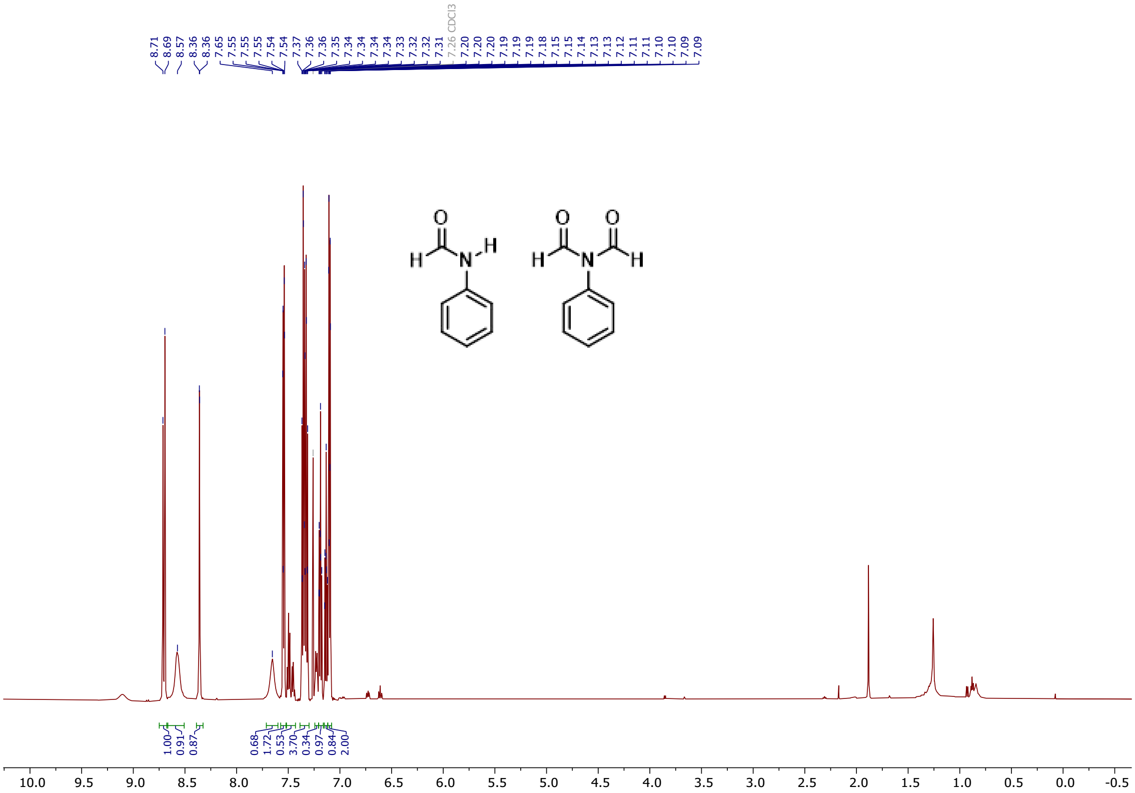

**Figure S29**. $^{1}H$ NMR spectrum (600 MHz, THF-$d_8$) of aniline N-formylation products (full window).

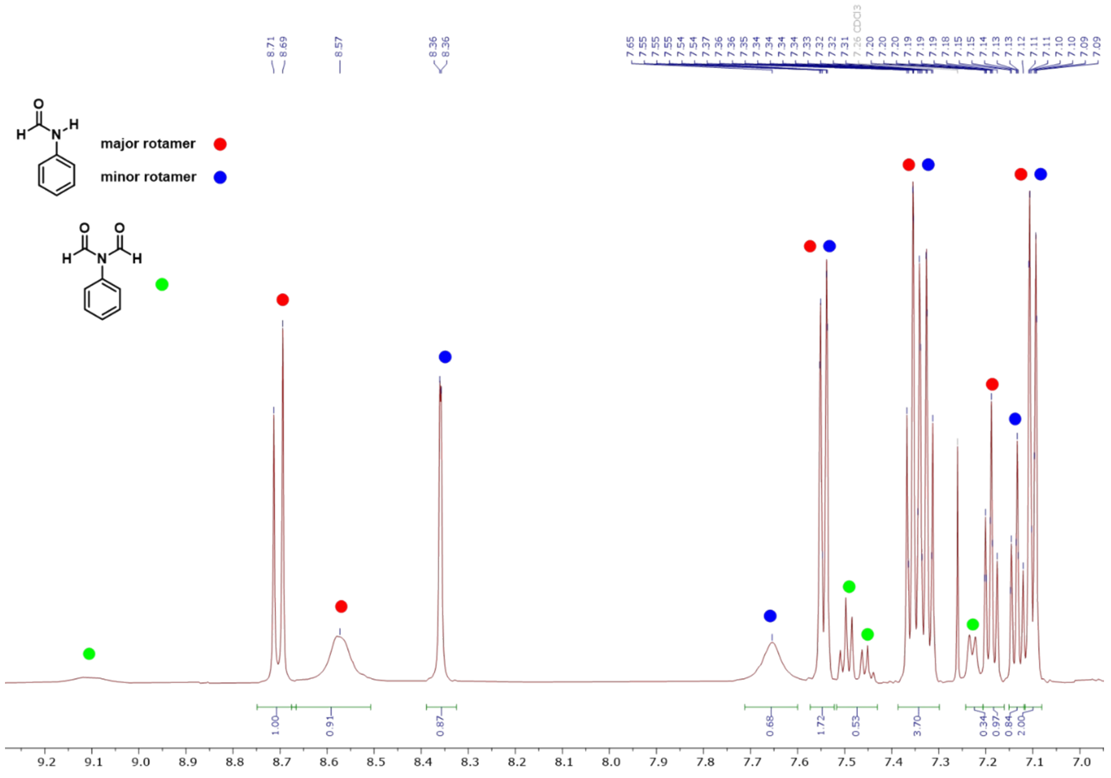

**Figure S30.** $^1$H NMR (600 MHz, THF-$d_8$) spectrum of N-formylation products.

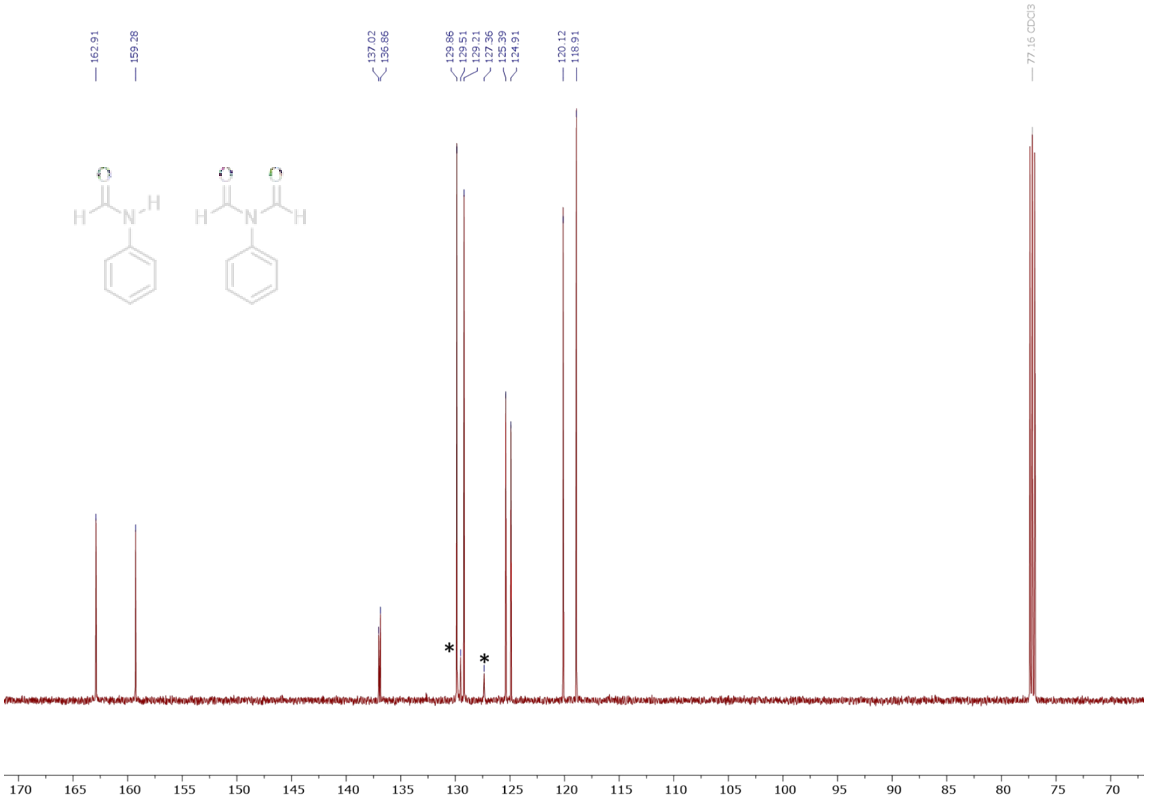

**Figure S31.** $^1$H NMR (75 MHz, THF-$d_8$) spectrum of N-formylation products. Asterisks denotes signals that belong to N-formyl-N-phenylformamide.

## IR-ATR Spectra

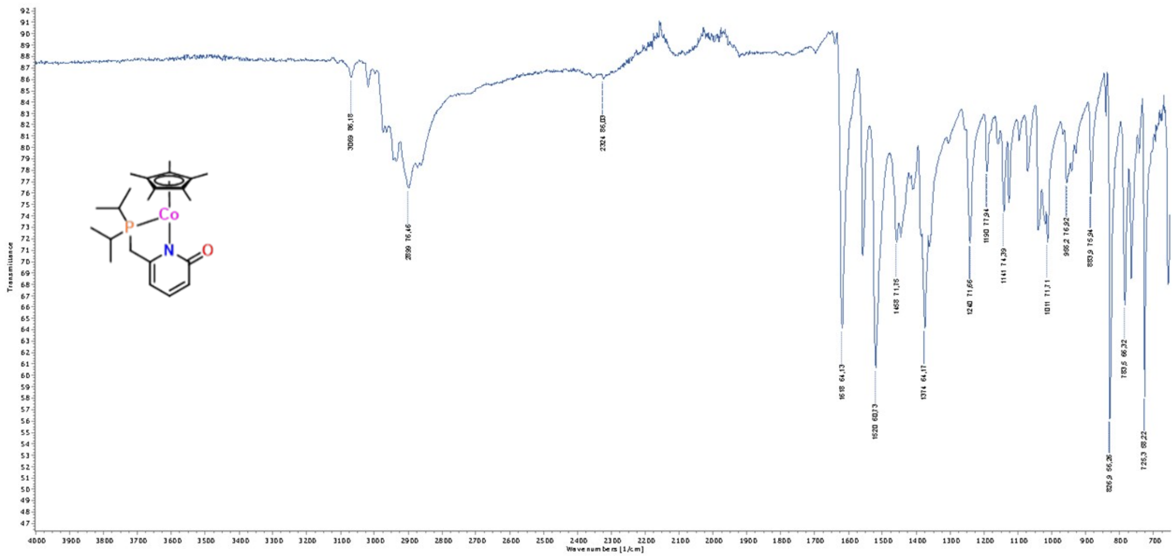

**Figure S32.** Transmission IR-ATR spectrum of **Co1**

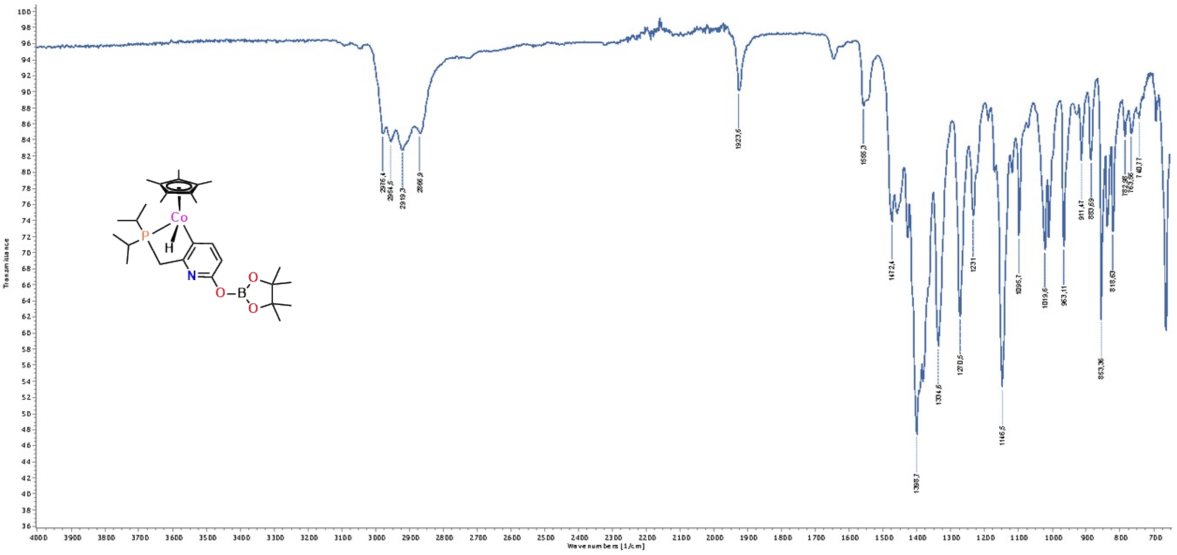

**Figure S33.** Transmission IR-ATR spectrum of **Co5**

## ESI-MS Analysis

*Sample preparation: A* crystalline sample of **Co1** was dissolved in THF and directly subjected to ESI-MS analysis; the mixture of **Co1** and HBpin (1.2 equiv.) was prepared *in situ* in THF at room temperature and directy subjected to ESI-MS analysis.
Sample solutions were transferred into a gas-tight syringe and fed into the ESI source of a micrOTOF-Q II mass spectrometer (Bruker Daltonik) at a flow rate of 0.5 mL $h^{-1}$. The ESI source was operated at a voltage of 4500 V with $N_2$ as nebulizer (8.0 psi backing pressure) and drying gas (heated to 333 K and held at 3.0 L $min^{-1}$ flow rate). The thus produced ions with 50 ≤ m/z ≤ 3000 were then allowed to pass the instrument's quadrupole mass filter and collision cell before entering the time-of-flight (TOF) mass analyzer. Ions were identified on the basis of their *m/z* ratio, their isotope pattern, and fragmentation patterns.

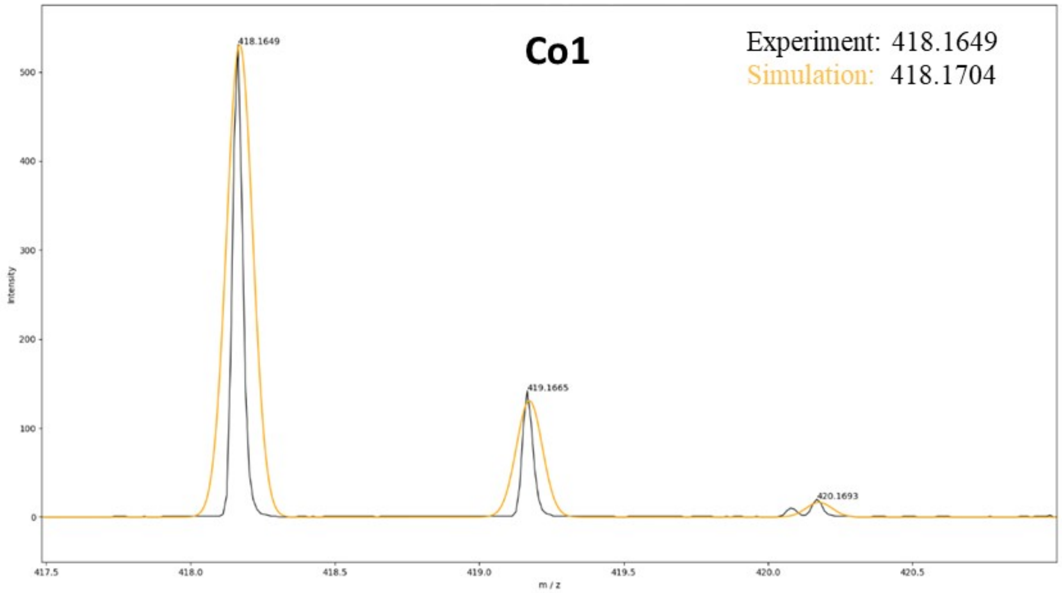


**Figure S34.** Fragment of positive-ion mode ESI mass spectrum of **Co1** in THF. Measured and simulated isotopic patterns depicted in black and orange respectively.

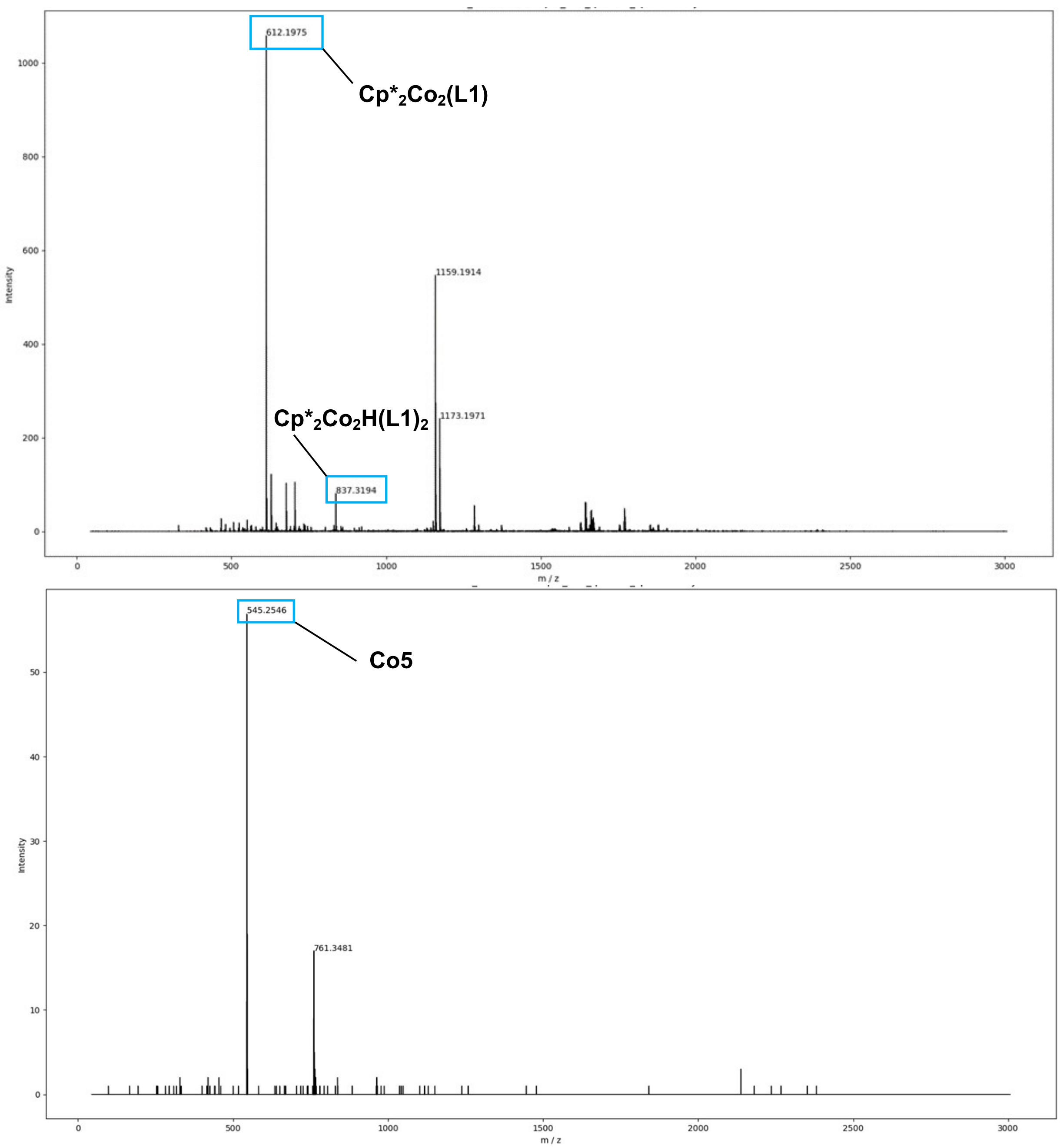


**Figure S35.** Positive-ion mode ESI mass spectra of a solution of the products formed in the reaction of **Co1** with HBpin (1.2 equiv.) in THF.

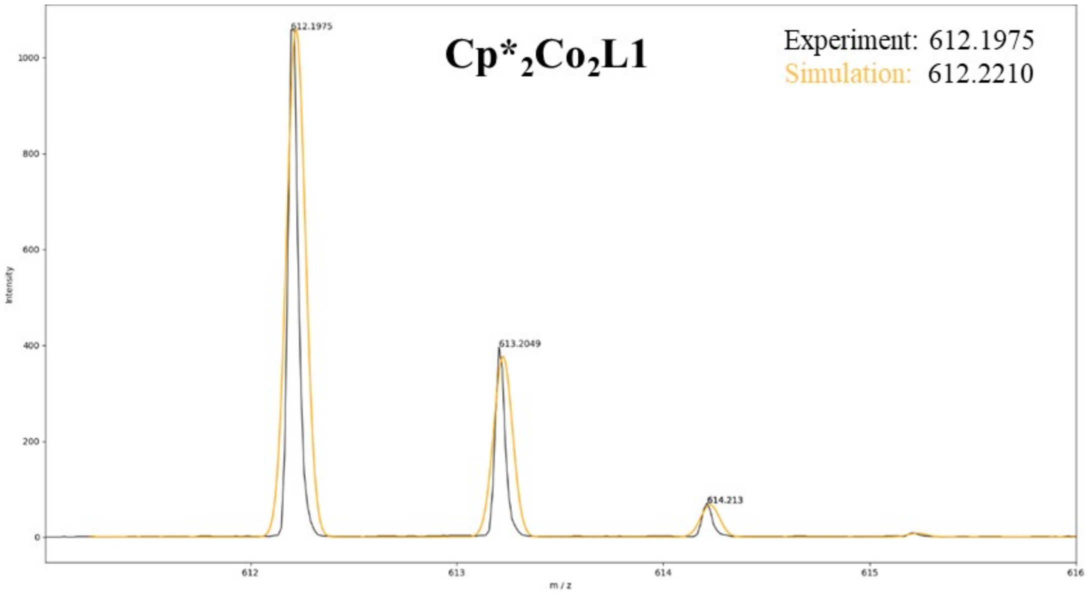


**Figure S36.** Measured (black) and simulated (orange) isotopic patterns of *in situ* generated Cp*$_2$Co$_2$(L1).

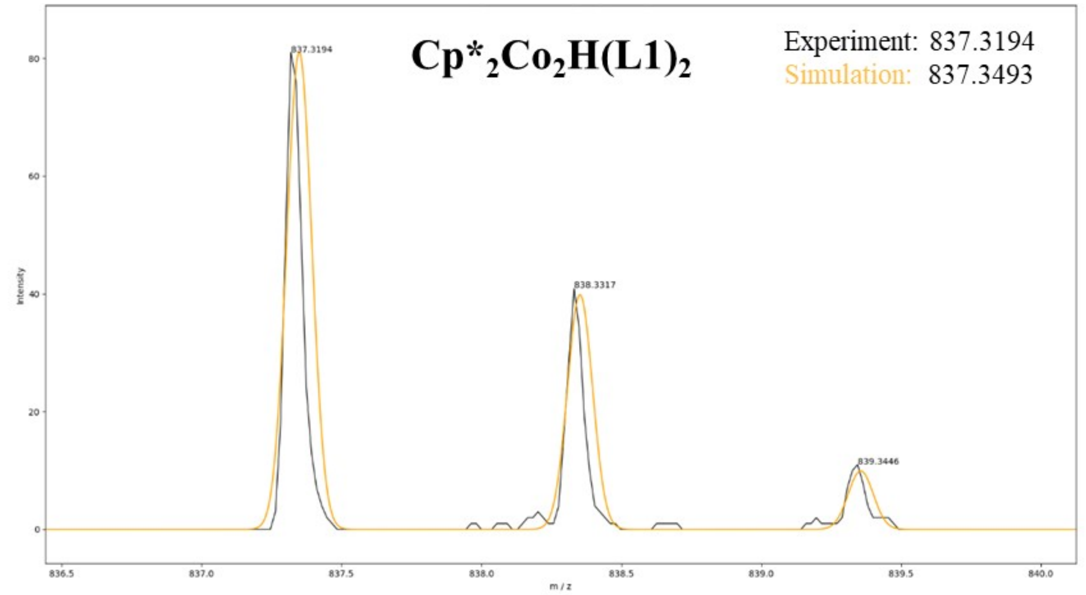


**Figure S37.** Measured (black) and simulated (orange) isotopic patterns of *in situ* generated Cp*$_2$Co$_2$H(L1)$_2$.

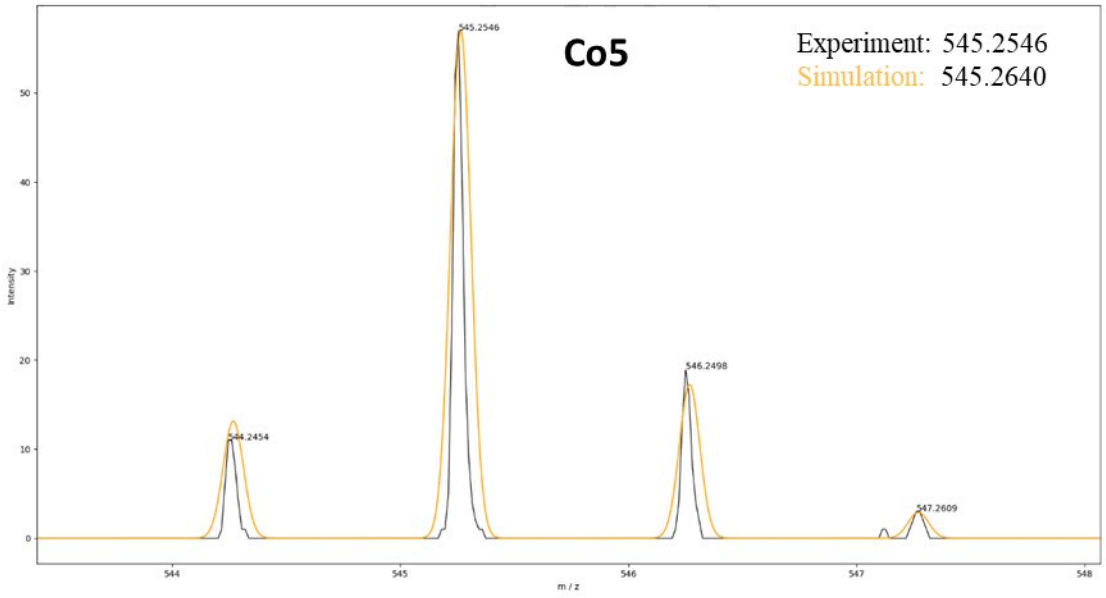


**Figure S38.** Measured (black) and simulated (orange) isotopic patterns of *in situ* generated **Co5**.

## X-ray Crystallography [6]

Single-crystal X-ray experiments were performed at 100 K using a SuperNova four-circle diffractometer in Kappa geometry with a 50 W Cu or Mo (Kα radiation) microfocus tube, an Atlas CCD detector (Rigaku Oxford Diffraction), and a Cryostream 700 Plus cooler (Oxford Cryosystems Ltd). Data collection, cell refinement, data reduction, and absorption correction were done using CrysAlisPro[22]. Intensities were measured using omega scans.

Single-crystal X-ray data was solved and refined as follows: The space group was determined either by using XPREP (Bruker AXS Inc.[23]) or CrysAlisPro and the phase problem was solved either (a) by structure-invariant direct methods with SHELXS[24], or (b) by using the dual-space algorithm implemented in SHELXT[25]. In every case, full-matrix least-squares refinement was done on $F^2$ using SHELXL[25].

Missing secondary atom sites were located from the difference Fourier map. If possible, non-hydrogen atoms were refined using individual, anisotropic displacement parameters. The fully refined data was reviewed using PLATON[26]. Carbon atom-bound hydrogen atoms were positioned geometrically and refined riding on their respective parent atoms. $U_{iso}$(H) was fixed at 1.5 ($CH_3$) or 1.2 (all other H atoms) of the parent atom's isotropic displacement parameter.

Both complexes **Co1** and **Co5** and their single crystal structure analyses were included in our recent report of a cobalt-catalyzed hydroboration of pyridines.[6]

**Table S1.** Crystal data and structure refinement for **Co1**.

| | |
|---|---|
| Identification code | Co1 |
| Empirical formula | $C_{22}H_{34}CoNOP$ |
| Formula weight | 418.40 |
| Temperature/K | 99.99(10) |
| Crystal system | orthorhombic |
| Space group | $Pna2_1$ |
| a/Å | 14.8126(2) |
| b/Å | 10.46320(10) |
| c/Å | 13.4959(2) |
| α/° | 90 |
| β/° | 90 |
| γ/° | 90 |
| Volume/Å$^3$ | 2091.69(5) |
| Z | 4 |
| $\rho_{calc}$g/cm$^3$ | 1.329 |
| μ/mm$^{-1}$ | 0.908 |
| F(000) | 892.0 |
| Crystal size/mm$^3$ | 0.32 × 0.25 × 0.2 |
| Radiation | Mo Kα (λ = 0.71073) |
| 2Θ range for data collection/° | 6.038 to 59.16 |
| Reflections collected | 88285 |
| Independent reflections | 5666 [$R_{int}$ = 0.0366, $R_{sigma}$ = 0.0142] |
| Data/restraints/parameters | 5666/1/244 |
| Goodness-of-fit on $F^2$ | 1.050 |
| Final R indexes [I>=2σ (I)] | $R_1$ = 0.0179, $wR_2$ = 0.0473 |
| Final R indexes [all data] | $R_1$ = 0.0183, $wR_2$ = 0.0475 |
| Largest diff. peak/hole / e Å$^{-3}$ | 0.25/-0.20 |
| Flack parameter | -0.003(2) |

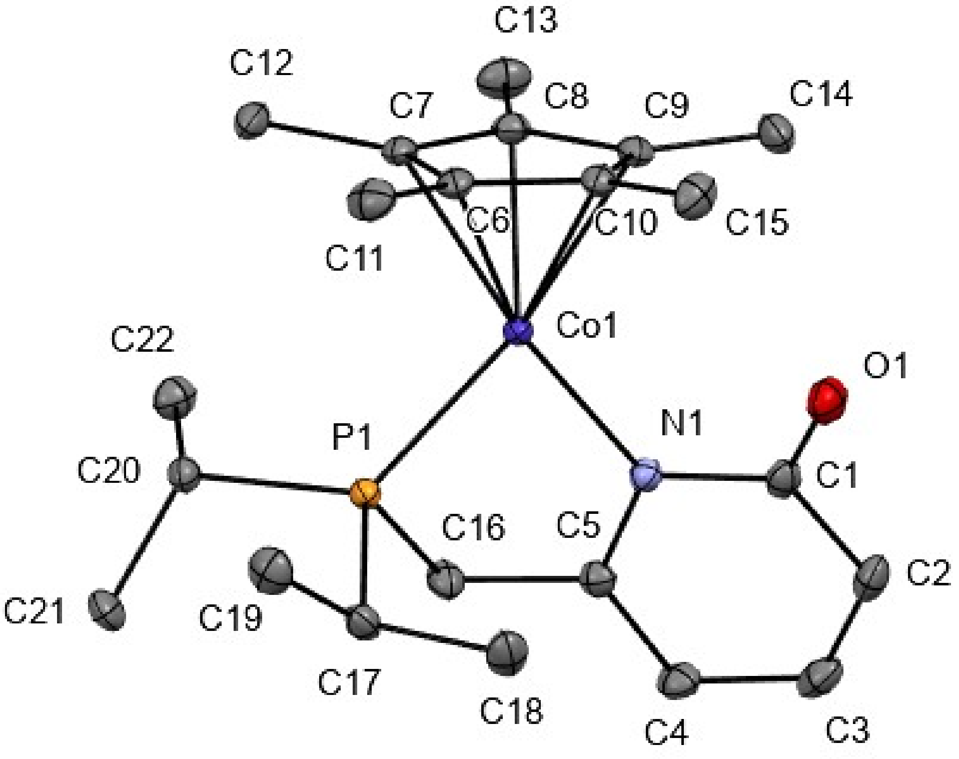


**Figure S39**. The molecular structure of **Co1.** Ellipsoids are shown at 50% probability. Hydrogen atoms are omitted for clarity.

**Table S2.** Crystal data and structure refinement for **Co5**.

| | |
|---|---|
| Identification code | Co5 |
| Empirical formula | $C_{28}H_{46}BCoNO_3P$ |
| Formula weight | 545.37 |
| Temperature/K | 99.97(13) |
| Crystal system | triclinic |
| Space group | P-1 |
| a/Å | 8.5688(5) |
| b/Å | 12.8423(5) |
| c/Å | 13.3857(5) |
| α/° | 100.810(3) |
| β/° | 95.593(4) |
| γ/° | 92.022(4) |
| Volume/Å$^3$ | 1437.81(12) |
| Z | 2 |
| $\rho_{calc}$g/cm$^3$ | 1.260 |
| μ/mm$^{-1}$ | 5.418 |
| F(000) | 584.0 |
| Crystal size/mm$^3$ | 0.24 × 0.14 × 0.08 |
| Radiation | CuKα (λ = 1.54184) |
| 2Θ range for data collection/° | 6.76 to 153.768 |
| Reflections collected | 28557 |
| Independent reflections | 5969 [$R_{int}$ = 0.0465, $R_{sigma}$ = 0.0310] |
| Data/restraints/parameters | 5969/0/325 |
| Goodness-of-fit on $F^2$ | 1.112 |
| Final R indexes [I>=2σ (I)] | $R_1$ = 0.0561, $wR_2$ = 0.1494 |
| Final R indexes [all data] | $R_1$ = 0.0611, $wR_2$ = 0.1588 |

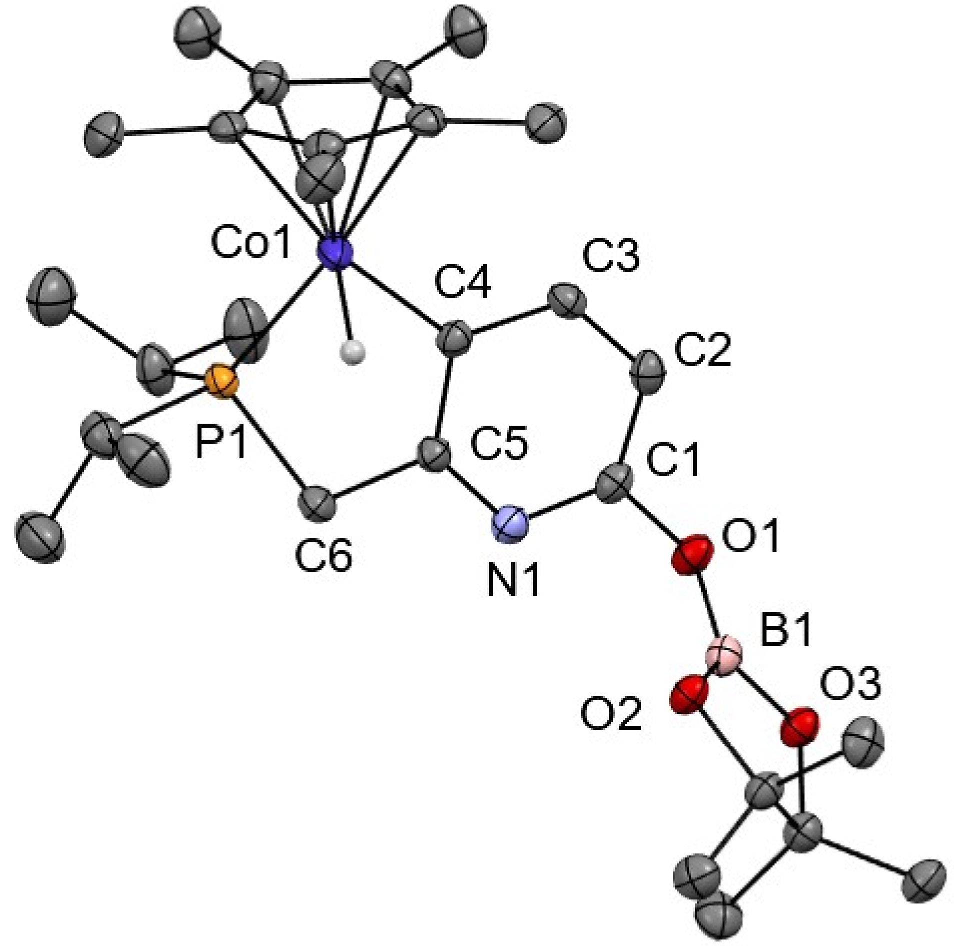


**Figure S40.** The molecular structure of **Co5.** Ellipsoids are shown at 50% probability. Hydrogen atoms are omitted for clarity (except of Co-H).

## Magnetic Measurements

Magnetic measurements for complex **Co1** were carried out with a Quantum-Design MPMS3 SQUID magnetometer equipped with a 7.0 T magnet. Direct current (dc) magnetic susceptibility measurements were performed under an applied dc field of 0.5 T with powdered polycrystalline samples packed in polycarbonate in a non-magnetic sample holder and covered with low viscosity perfluoropolyether-based inert oil Fomblin Y45 in the range from 200.0 K to 2.0 K (the upper temperature limit was chosen because of the pour point of the oil). The oil was dried under vacuum and degassed prior to use. Sample preparation was performed inside a glove box under $N_2$ atmosphere. Each raw data of the measured magnetic moment was corrected for the diamagnetic contribution of the capsules according to $M_{dia}$(capsule) = $\chi_g \cdot m \cdot H$, with an experimentally obtained gram susceptibility of the capsules including the inert oil. The diamagnetic contribution of the compounds was corrected using Pascal's constants. Alternating current (ac) susceptibility measurements were carried out in an oscillating ac field of 3.0 Oe and frequencies ranging from 0.1 to 1000 Hz.

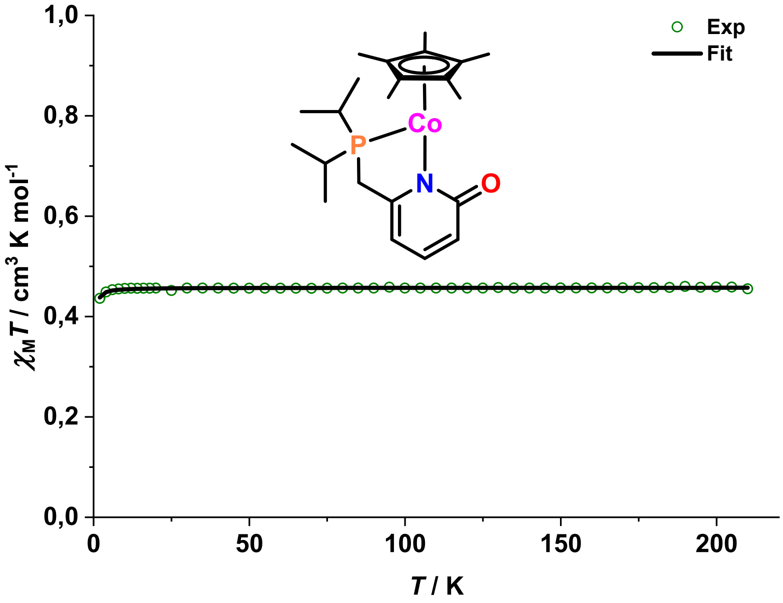


**Figure S41.** Temperature dependence of $\chi_M T$ product for the mononuclear Co(II) complex measured under an applied dc field of 0.5 T

Low-spin Co(II) complex, $S = ½$, $g = 2.21$, [illegible] = - 0.07 K (intermolecular interaction), TIP = 157 x $10^{-6}$ cm$^3$ mol$^{-1}$

## Literature Overview of Catalysts and Reaction Condition in Hydroboration of $CO_2$

**Table S3.** Overview of <u>selected</u> literature-reported catalysts and reaction conditions in the hydroboration of $CO_2$ to the formate level.

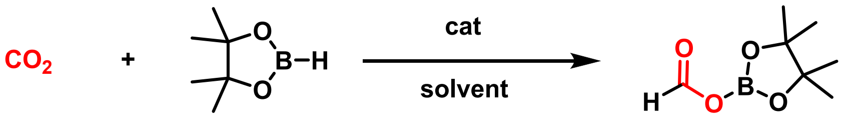


| Entry | Catalyst | cat. loading (mol%) | Solvent | Temp °C | Time | Additives | Yield | Best TON/TOF($h^{-1}$) | Ref |
|---|---|---|---|---|---|---|---|---|---|
| 1 | 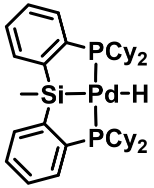 | 0.01 | $C_6D_6$ | rt | 1 h | none | 85 | 8500/8500 | [12] |
| | | 0.001 | $C_6D_6$ | rt | 120 h | none | 64 | 63500/530 | |
| 2 | 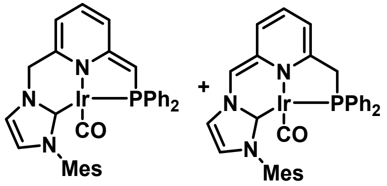 | 0.1 | THF-$d_8$ | 30 | 1 h | $H_2O$ (3 mol%) | 74 | 740/740 | [13] |
| | | 0.2 | THF-$d_8$ | 30 | 20 min | $H_2O$ (7 mol%) | 83 | 415/1245 | |
| 3 | 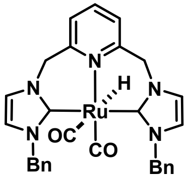 | 1.0 | $CH_2Cl_2$ | rt | 30 min | KO$^t$Bu (2.5mol%) | 70 | 70/140 | [14] |
| | | 1.0 | $CH_2Cl_2$ | rt | 30 min | KOCO$_2$$^t$Bu (2.5 mol%) | 76 | 76/152 | |

| Entry | Catalyst | cat. loading (mol%) | Solvent | Temp °C | Time | Additives | Yield | Best TON/TOF($h^{-1}$) | Ref |
|---|---|---|---|---|---|---|---|---|---|
| 4* | 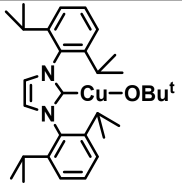 | 10 mol% | THF | 35 | 24 h | none | 85 | 8.5/0.4 | [15] |
| 5 | 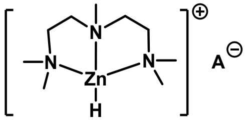 A = $[B(C_6F_5)_4]$ or $[B(3,5\text{-}Me_2\text{-}C_6H_3)_4]$ | 1.0 mol% | $CH_3CN\text{-}d_3$ | 60 | 2 h | none | 94 | 94/42 | [16] |
| 6 | 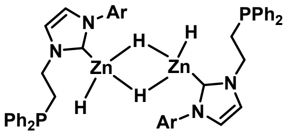 Ar = DIPP | 5.0 mol% | $C_6D_6$ | rt | 12 h | none | 85 | 17/1.4 | [17] |
| 7 | 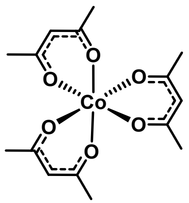 | 1.0 mol % | $THF\text{-}d_8$ | 50 | 16 h | $NaHBEt_3$ (1.0 mol%) | 27 | 27/1.7 | [7] |
| 8 | 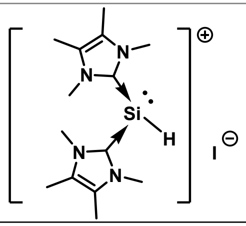 | 10.0 | $C_6D_6$ | 90 | 10 min | none | 98 | 9.8/58.8 | [18] |

| Entry | Catalyst | cat. loading (mol%) | Solvent | Temp °C | Time | Additives | Yield | Best TON/TOF($h^{-1}$) | Ref |
|---|---|---|---|---|---|---|---|---|---|
| 9 | 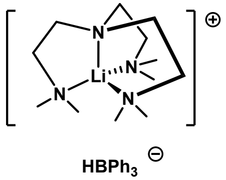 | 1.0 | THF-$d_8$ | 25 | 10 h | none | 100 | 100/10 | [19] |
| 10 | 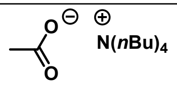 | 5.0 | $C_6D_6$ | 90 | 2 h | none | 62 | 12.4/6.2 | [20] |
| 11 | 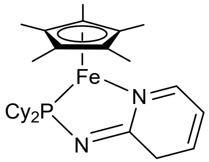 | 1.0 | $C_6D_6$ | 25 | 5 min | none | 98 | 98/1176 | [21] |

* yield of formic acid after hydrolysis

## References


[1] S. D. Toto, J. T. Doi, *J. Org. Chem.* **1987**, *52*, 4999–5003.

[2] F. Jiang, M. Achard, T. Roisnel, V. Dorcet, C. Bruneau, *Eur. J. Inorg. Chem.* **2015**, *2015*, 4312–4317.

[3] U. Kölle, F. Khouzami, B. Fuss, *Angew. Chem. Int. Ed.* **1982**, *21*, 230–240.

[4] X. Zhuang, J. Y. Chen, Z. Yang, M. Jia, C. Wu, R. Z. Liao, C. H. Tung, W. Wang, *Organometallics* **2019**, *38*, 3752–3759.

[5] S. K. Sur, *J. Magn. Reson.* **1989**, *82*, 169–173.

[6] F. Hoeeg, L. Luxenberger, A. Fedulin, A. Jacobi von Wangelin, *Chem. Sci.* **2024**,15, 5201-5210.

[7] S. R. Tamang, M. Findlater, *Dalton Trans.* **2018**, *47*, 8199–8203.

[8] C. Erken, A. Kaithal, S. Sen, T. Weyhermüller, M. Hölscher, C. Werlé, W. Leitner, *Nat. Commun.* **2018**, *9*, 1–9.

[9] X.-T. Lin, K. Matsumoto, Y. Maegawa, K. Takeuchi, N. Fukaya, K. Sato, S. Inagaki, J.-C. Choi, *New J. Chem.* **2021**, *45*, 9501–9505.

[10] P. Nad, K. Gupta, A. Sen, A. Mukherjee, *Chem. Asian J.* **2022**, *17*, e202200800.

[11] M. R. I. I. I. Smith, R. Bisht, C. Haldar, G. Pandey, J. E. Dannatt, B. Ghaffari, R. E. J. Maleczka, B. Chattopadhyay, *ACS Catal.* **2018**, *8*, 6216–6223.

[12] a) H. W. Suh, L. M. Guard, N. Hazari, *Chem. Sci.* **2014**, *5*, 3859–3872. b) M. R. Espinosa, D. J. Charboneau, A. Garcia De Oliveira, N. Hazari, *ACS Catal.* **2019**, 9, 301.

[13] P. Sánchez, M. Hernández-Juárez, N. Rendón, J. López-Serrano, E. Álvarez, M. Paneque, A. Suárez, *Dalton Trans.* **2018**, *47*, 16766–16776.

[14] C. K. Ng, J. Wu, T. S. A. Hor, H. K. Luo, *Chem. Commun.* **2016**, *52*, 11842–11845.

[15] R. Shintani, K. Nozaki, *Organometallics* **2013**, *32*, 2459–2462.

[16] R. Chambenahalli, R. M. Bhargav, K. N. McCabe, A. P. Andrews, F. Ritter, J. Okuda, L. Maron, A. Venugopal, *Chem. Eur. J.* **2021**, *27*, 7391–7401.

[17] X. Wang, K. Chang, X. Xu, *Dalton Trans.* **2020**, *49*, 7324–7327.

[18] B. X. Leong, J. Lee, Y. Li, M. C. Yang, C. K. Siu, M. Der Su, C. W. So, *J. Am. Chem. Soc.* **2019**, *141*, 17629–17636.

[19] D. Mukherjee, H. Osseili, T. P. Spaniol, J. Okuda, *J. Am. Chem. Soc.* **2016**, *138*, 10790–10793.

[20] Y. C. A. Sokolovicz, O. Nieto Faza, D. Specklin, B. Jacques, C. S. López, J. H.

Z. Dos Santos, H. S. Schrekker, S. Dagorne, *Catal. Sci. Technol.* **2020**, *10*, 2407–2414.

[21] H. Gao, J. Jia, C.-H. Tung, W. Wang, *Organometallics* **2023**, *42*, 944–951.

[22] Agilent Technologies Ltd, Yarnton, Oxfordshire, England, **2014**.

[23] Bruker AXS Inc., Madison, Wisconsin, USA, **2014**.

[24] G. M. Sheldrick, *Acta Crystallogr. A* **2008**, *64*, 112–122.

[25] G. M. Sheldrick, *Acta Crystallogr. A* **2015**, *71*, 3–8.

[26] A. L. Spek, *Acta Crystallogr. D* **2009**, *65*, 148–55.
